# Transcriptomics analysis of differentially expressed genes in subcutaneous and perirenal adipose tissue of sheep as affected by their pre- and early postnatal malnutrition histories

**DOI:** 10.1186/s12864-021-07672-5

**Published:** 2021-05-11

**Authors:** Sharmila Ahmad, Markus Hodal Drag, Suraya Mohamad Salleh, Zexi Cai, Mette Olaf Nielsen

**Affiliations:** 1 Nutrition Research Unit, Department of Animal Science, Aarhus University, Blichers Alle 20, 8830 Tjele, Denmark; 2 Novo Nordisk Foundation Center for Basic Metabolic Research, Faculty of Health and Medical Sciences, University of Copenhagen, Blegdamsvej 3B, 2200 Copenhagen, Denmark; 3 Department of Animal Science, Universiti Putra Malaysia, 43400 Serdang, Selangor Malaysia; 4 Department of Animal Nutrition and Management, Swedish University of Agricultural Sciences, 750 07 Uppsala, Sweden; 5 Centre for Quantitative Genetics and Genomics, Aarhus University, Blichers Alle 20, 8830 Tjele, Denmark

**Keywords:** Early life malnutrition, Subcutaneous adipose tissue, Perirenal adipose tissue, Differential expressed genes, Functional enrichment, Long-term programming

## Abstract

**Background:**

Early life malnutrition is known to target adipose tissue with varying impact depending on timing of the insult. This study aimed to identify differentially expressed genes in subcutaneous (SUB) and perirenal (PER) adipose tissue of 2.5-years old sheep to elucidate the biology underlying differential impacts of late gestation versus early postnatal malnutrition on functional development of adipose tissues. Adipose tissues were obtained from 37 adult sheep born as twins to dams fed either NORM (fulfilling energy and protein requirements), LOW (50% of NORM) or HIGH (110% of protein and 150% of energy requirements) diets in the last 6-weeks of gestation. From day 3 to 6 months of age, lambs were fed high-carbohydrate-high-fat (HCHF) or moderate low-fat (CONV) diets, and thereafter the same moderate low-fat diet.

**Results:**

The gene expression profile of SUB in the adult sheep was not affected by the pre- or early postnatal nutrition history. In PER, 993 and 186 differentially expressed genes (DEGs) were identified in LOW versus HIGH and NORM, respectively, but no DEG was found between HIGH and NORM. DEGs identified in the mismatched pre- and postnatal nutrition groups LOW-HCHF (101) and HIGH-HCHF (192) were largely downregulated compared to NORM-CONV. Out of 831 DEGs, 595 and 236 were up- and downregulated in HCHF versus CONV, respectively. The functional enrichment analyses revealed that transmembrane (ion) transport activities, motor activities related to cytoskeletal and spermatozoa function (microtubules and the cytoskeletal motor protein, dynein), and responsiveness to the (micro) environmental extracellular conditions, including endocrine and nervous stimuli were enriched in the DEGs of LOW versus HIGH and NORM. We confirmed that mismatched pre- and postnatal feeding was associated with long-term programming of adipose tissue remodeling and immunity-related pathways. In agreement with phenotypic measurements, early postnatal HCHF feeding targeted pathways involved in kidney cell differentiation, and mismatched LOW-HCHF sheep had specific impairments in cholesterol metabolism pathways.

**Conclusions:**

Both pre- and postnatal malnutrition differentially programmed (patho-) physiological pathways with implications for adipose functional development associated with metabolic dysfunctions, and PER was a major target.

**Supplementary Information:**

The online version contains supplementary material available at 10.1186/s12864-021-07672-5.

## Background

Compromised nutrition during fetal life may alter the growth trajectory of many developing organs, including adipose tissues, due to a phenomenon termed fetal programming. This can lead to tissue malfunction and development of health disorders later in life [1, 2]. In addition to genetic modifications, such nutrient-regulated gene expression may play a major role in the development of adult disease [3].

Fat distribution patterns in the body, capacity of adipose tissues to accommodate nutrient excess, and fat cell size distribution patterns, rather than total fat mass, are major determinant risk factors for predisposition of metabolic disarrangements [4–6]. Subcutaneous adipose tissue (SUB) plays a key role in fat partitioning by preventing nutrient overflow and hence fat deposition elsewhere (e.g. abdominal fats and non-adipose tissue) [7–9], whereas central obesity and ectopic fat deposition are well-known risk factors for insulin resistance and cardiovascular diseases [10, 11]. In contrast to SUB, the specific roles of perirenal adipose tissue (PER) in relation to development of obesity and associated disorders is less elucidated, and most studies of PER in humans have relied on indirect measurements using ultrasound and other non-invasive approaches [12]. Nevertheless, perirenal fat thickness was shown to be a determining factor for kidney dysfunction and correlated to risk of severe kidney disease and hypertension in humans [13, 14].

We have previously shown in a sheep model that late gestation and early postnatal malnutrition can induce differential, depot and sex-specific changes in adipose developmental traits and metabolic outcomes in adulthood, with PER and SUB as the major targets of prenatal programming in contrast to mesenteric and epicardial adipose tissue [15, 16]. Moreover, in our sheep model we observed a 1/3 reduction in kidney weight of adolescent sheep that had been exposed to an obesogenic high-carbohydrate-high-fat (HCHF) diet in early postnatal life [17]. Furthermore, sheep with a history of prenatal undernutrition followed by early postnatal obesity development developed hypercholesterolemia, which persisted into adulthood even after 2 years of dietary correction [16, 17].

Gene expression profiling studies of adipose tissue have revealed vast numbers of different adipose molecular markers, especially inflammatory genes, that could be linking expanded fat mass and obesity co-morbidities [18]. In this context, nutrition has been shown to program gene expression and development of adipose tissue (i.e. SUB, PER and omental fat) in different animal models [19–22]. Concordantly, using quantitative real-time polymerase chain reaction (qPCR) analysis, we have previously documented impacts of late gestation and early postnatal nutrition interventions on gene expression of well-known markers for adipose development, adipose metabolisms as well as inflammation in four different adipose tissues (SUB, PER, mesenteric and epicardial) of 6-months old lambs and 2.5-years old sheep. However, the early nutrition impacts on gene expression for these markers were poorly associated to observed changes in adipose morphology, cellularity and cell size distributions [15, 23].

In this study, we therefore aimed to unravel the genes and/or pathways responsible for the observed changes in adipose morphological traits and the phenotypic manifestations observed in adipose tissue of these animals by using a transcriptomic analysis approach. Application of RNA-sequencing and transcriptomic methodologies could reveal underlying hitherto unknown pathways involved in tissue specific responses to early malnutrition, leading to identification of potential candidate markers for fetal programming (hub genes) and shedding light on the involvement of different adipose tissues in organ and metabolic dysfunctions arising from adverse programming in early life.

## Results

### Mapping summary

A total of 67 samples (SUB = 31 and PER = 36, respectively) were analyzed using RNA-sequencing. After filtering, the mean numbers of clean reads per sample obtained from SUB and PER were 34,137,165 and 34,818,481, respectively, and were aligned against *Ovis aries* reference genome (oar_v3.1) using the software package STAR. On average, 83% of the total reads were successfully mapped allowing no more than eight mismatches and restricting the alignments at most 40 genomic locations. Among the aligned reads, approximately 86 and 65% were mapped to unique genomic regions in SUB and PER, respectively. The mean coverages of paired-end reads mapping to exonic, intronic, intergenic, and intronic/intergenic regions were 26.48, 36.54, 36.98 and 7.28% for SUB and 19.22, 40.55, 40.22 and 6.01% for PER, respectively.

### Differentially expressed genes (DEGs)

**Fig. 1:**
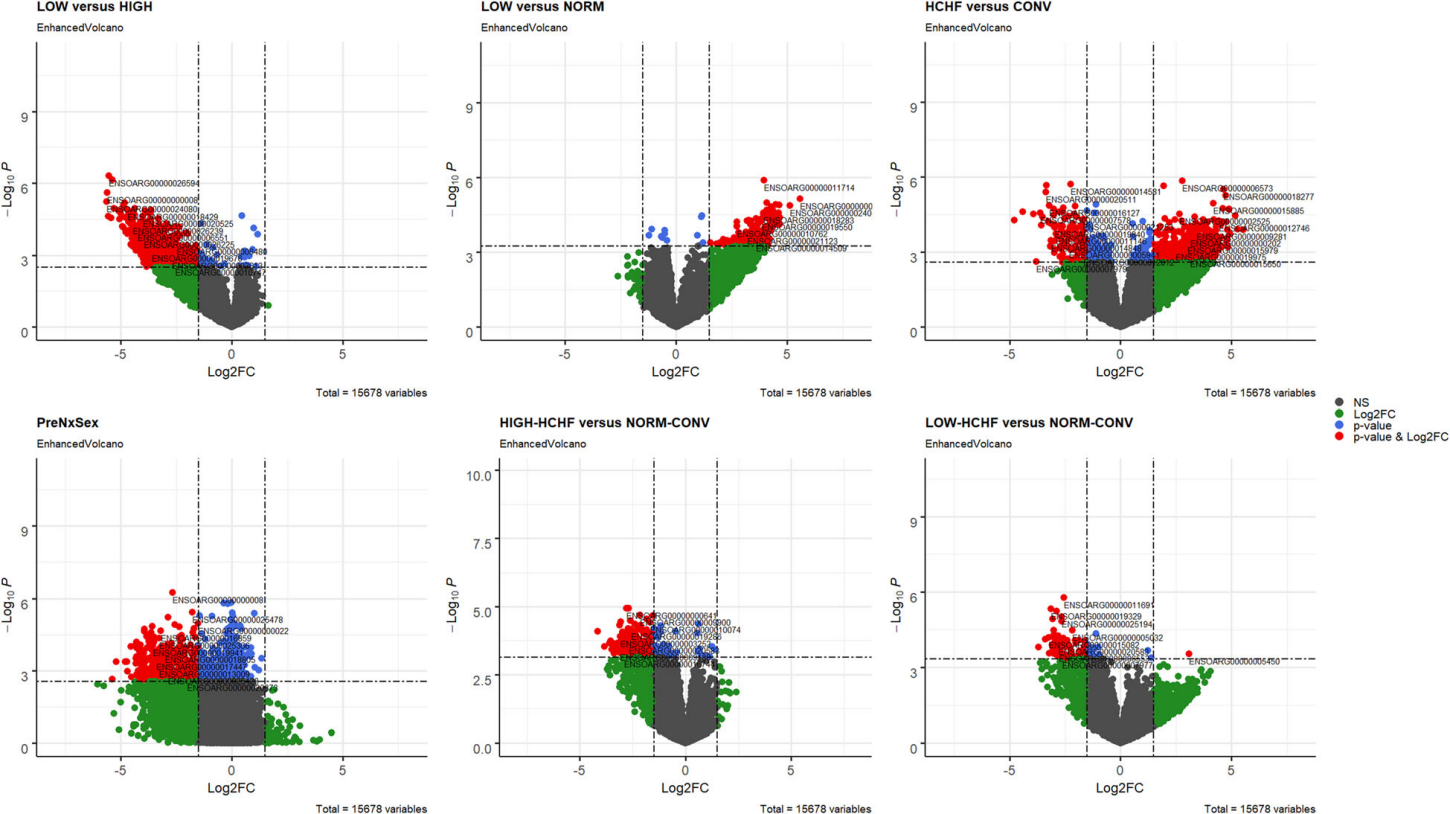
Volcano plots depicting the log2 Fold changes for gene expression levels between different groups. Sheep with different pre- (NORM, LOW, HIGH) and postnatal (CONV, HCHF) nutrition histories. **a** LOW vs HIGH, **b** LOW vs NORM, **c** interaction effect of prenatal nutrition and sex (PreNxsex), **d** LOW-HCHF vs NORM-CONV, **e** HIGH-HCHF vs NORM-CONV, and **f** HCHF vs CONV. Red and green dots indicate genes up- or downregulated with more or less than 1.50 or − 1.50 fold change, respectively, with padj < 0.05. Genes with black dots were not significantly differentially expressed. The blue dots represent the padj < 0.05

The lists of differential expressed genes (DEGs) after Benjamini-Hochberg correction, padj < 0.05 are shown in Table 1, and the direction of change of expression for DEGs for each group comparison are shown in Fig. 1. The gene expression profiles of SUB in the adult sheep were not affected by the pre- or early postnatal nutrition history or sex, except for 44 DEGs identified (padj < 0.05) between adult males and females (Additional file 1: Supplementary Table 1).

**Table 1:** The number of up- and downregulated DEGs for different group comparison

No.	Pairwise contrast	Total DEGs	Upregulated	Downregulated
1	LOW vs HIGH	993	18	975
2	LOW vs NORM	186	179	7
3	Prenatal nutrition x sex (PreNxsex)	873	171	702
4	LOW-HCHF vs NORM-CONV	101	1	100
5	HIGH-HCHF vs NORM-CONV	192	12	180
6	HCHF vs CONV	831	595	236

In PER, 993 DEGs were identified in LOW sheep compared to HIGH, of which 975 and 18 genes were down- and upregulated, respectively. Of the known downregulated DEGs, 87 had a fold change (FC) between − 4.00 and − 5.50, whereas the FC for the upregulated DEGs was ranging from 0.10 to 0.30. In LOW vs NORM sheep, 179 upregulated and 7 downregulated DEGs were identified, of which (for the known upregulated DEGs) 25 had an FC > 4.00. The likelihood ratio test for the interaction between prenatal nutrition and sex revealed 869 out of 873 DEGs were downregulated. There were no DEG identified between HIGH and NORM.

DEG analysis was also done between six combinations of pre- and postnatal nutrition, namely NORM-CONV, NORM-HCHF, LOW-CONV, LOW-HCHF, HIGH-CONV, and HIGH-HCHF. Among them, a total of 101 and 192 genes showed significant (padj < 0.05) differential expression between LOW-HCHF vs NORM-CONV and between HIGH-HCHF vs NORM-CONV, respectively. No DEGs were identified for the other group comparisons. In particular, 100 out of 101 and 180 out of 192 genes were downregulated in LOW-HCHF and HIGH-HCHF compared to NORM-CONV, respectively. Of the known downregulated DEGs in LOW-HCHF vs NORM-CONV, 9 had an FC < -3.00, and for HIGH-HCHF vs NORM-CONV, 14 had an FC < -3.00.

For the independent effect of early postnatal nutrition, 831 DEGs were identified with 595 upregulated and 236 downregulated in HCHF compared to CONV sheep. Of the known upregulated DEGs, 50 had a FC between 4.00 and 5.50, whereas for downregulated DEGs, 13 had a FC between − 3.00 to − 5.00. The list of the top 20 known up- and downregulated DEGs for all the group comparison, ranked by log2 Fold Change (log2FC), are shown in Table 2.

**Table 2:** The top 10 known up- and downregulated DEGs for the six (A-F) group comparisons

Gene symbol	Log2FC	*P*-value	padj	Encoded protein	Expression
A) LOW vs HIGH					
*DSCAM*	−5.460	2.84E-05	0.015	Down Syndrome Cell Adhesion Molecule	down
*C10orf71*	−5.395	7.26E-07	0.006	Chromosome 10 Open Reading Frame 71	down
*TMEM63C*	−5.120	1.42E-05	0.015	Transmembrane Protein 63C	down
*FAM221A*	−4.924	6.54E-05	0.015	Family With Sequence Similarity 221 Member A	down
*ADAM7*	−4.889	1.08E-05	0.0149	ADAM Metallopeptidase Domain 7	down
*STRC*	−4.876	7.30E-05	0.016	Stereocilin	down
*TKTL1*	−4.824	6.83E-06	0.015	Transketolase Like 1	down
*CYP24A1*	−4.770	0.0001	0.0167	Cytochrome P450 Family 24 Subfamily A Member 1	down
*MYT1*	−4.708	0.0001	0.017	Myelin Transcription Factor 1	down
*ARHGEF5*	−4.703	1.30E-05	0.015	Rho Guanine Nucleotide Exchange Factor 5	down
*ETFRF1*	0.997	7.44E-05	0.015	Electron Transfer Flavoprotein Regulatory Factor 1	up
*H1–0*	0.947	0.0006	0.023	H1.0 Linker Histone	up
*CHCHD1*	0.790	0.001	0.028	Coiled-Coil-Helix-Coiled-Coil-Helix Domain Containing 1	up
*PECR*	0.689	0.003	0.048	Peroxisomal Trans-2-Enoyl-CoA Reductase	up
*FBXO6*	0.685	0.001	0.027	F-Box Protein 6	up
*ARL2*	0.642	0.003	0.044	ADP Ribosylation Factor Like GTPase 2	up
*MRPL20*	0.625	0.0007	0.024	Mitochondrial Ribosomal Protein L20	up
*WARS1*	0.576	0.001	0.030	Tryptophanyl-TRNA Synthetase 1	up
*PSMB9*	0.574	0.0007	0.023	Proteasome 20S Subunit Beta 9	up
*SNAPIN*	0.560	0.003	0.046	SNAP Associated Protein	up
B) LOW vs NORM					
*CADM1*	−1.234	0.0002	0.041	Cell Adhesion Molecule 1	down
*CXCR4*	−1.097	0.0001	0.036	C-X-C Motif Chemokine Receptor 4	down
*VRK2*	−0.678	0.0002	0.042	VRK Serine/Threonine Kinase 2	down
*GXYLT1*	−0.617	0.0003	0.043	Glucoside Xylosyltransferase 1	down
*SELENOI*	−0.519	0.0002	0.039	Selenoprotein I	down
*TRMT13*	−0.511	0.0001	0.036	TRNA Methyltransferase 13 Homolog	down
*PIKFYVE*	−0.419	0.0004	0.043	Phosphoinositide Kinase, FYVE-Type Zinc Finger Containing	down
*FSIP2*	4.677	4.09E-05	0.026	Fibrous Sheath Interacting Protein 2	up
*XDH*	4.661	7.57E-05	0.030	Xanthine Dehydrogenase	up
*IQCH*	4.631	1.33E-05	0.026	IQ Motif Containing H	up
*DCST1*	4.630	8.15E-05	0.031	DC-STAMP Domain Containing 1	up
*LIPN*	4.557	3.09E-05	0.026	Lipase Family Member N	up
*CSMD3*	4.452	6.03E-05	0.027	CUB And Sushi Multiple Domains 3	up
*ABCA12*	4.450	4.66E-05	0.026	ATP Binding Cassette Subfamily A Member 12	up
RN*F17*	4.409	3.85E-05	0.026	Ring Finger Protein 17	up
*ANO4*	4.370	9.46E-05	0.032	Anoctamin 4	up
*CPN1*	4.323	3.01E-05	0.026	Carboxypeptidase N Subunit 1	up
C) PreNxsex					
*TEX11*	−5.219	0.0004	0.023	Testis Expressed 11	down
*FAT2*	−4.728	0.0004	0.023	FAT Atypical Cadherin 2	down
*FSIP2*	−4.695	0.001	0.034	Fibrous Sheath Interacting Protein 2	down
*CCDC180*	−4.534	0.002	0.042	Coiled-Coil Domain Containing 180	down
*ROBO3*	−4.324	0.0005	0.024	Roundabout Guidance Receptor 3	down
*LIPN*	−4.319	8.00E-05	0.018	Lipase Family Member N	down
*ABCA12*	−4.286	0.0001	0.018	ATP Binding Cassette Subfamily A Member 12	down
*CNTNAP5*	−4.220	0.002	0.042	Contactin Associated Protein Family Member 5	down
*CSMD3*	−4.194	0.0001	0.018	CUB And Sushi Multiple Domains 3	down
*SLC26A5*	−4.157	0.0001	0.018	Solute Carrier Family 26 Member 5	down
*ANKS4B*	1.046	0.0007	0.029	Ankyrin Repeat And Sterile Alpha Motif Domain Containing 4B	up
*EPHA2*	0.880	0.0002	0.019	EPH Receptor A2	up
*PTMA*	0.820	0.0002	0.020	Prothymosin Alpha	up
*BTBD19*	0.767	0.0002	0.019	BTB Domain Containing 19	up
*EGR2*	0.660	0.001	0.036	Early Growth Response 2	up
*FBRS*	0.630	0.0001	0.018	FAU Ubiquitin Like And Ribosomal Protein S30 Fusion	up
*MAPK8IP3*	0.588	0.0006	0.026	Mitogen-Activated Protein Kinase 8 Interacting Protein 3	up
*PLEKHG2*	0.568	0.001	0.036	Pleckstrin Homology And RhoGEF Domain Containing G2	up
*SYNDIG1L*	0.567	0.0003	0.021	Synapse Differentiation Inducing 1 Like	up
*ARSL*	0.560	0.0003	0.022	Arylsulfatase L	up
D) LOW-HCHF vs NORM-CONV					
*LIPA*	−3.246	6.49E-05	0.040	Lipase A, Lysosomal Acid Type	down
*FN1*	−3.124	4.58E-06	0.021	Fibronectin 1	down
*ITGB2*	−3.094	0.0001	0.040	Integrin Subunit Beta 2	down
*LGALS3*	−3.054	1.21E-05	0.031	Galectin 3	down
*MSR1*	−3.035	0.0002	0.041	Macrophage Scavenger Receptor 1	down
*MS4A8*	− 3.025	0.0002	0.041	Membrane Spanning 4-Domains A8	down
*LCP1*	− 3.029	5.66E-05	0.040	Lymphocyte Cytosolic Protein 1	down
*CAPG*	−3.020	0.0002	0.044	Capping Actin Protein, Gelsolin Like	down
*CTSZ*	−3.011	3.40E-05	0.040	Cathepsin Z	down
*C5AR1*	−2.985	0.0002	0.040	Complement C5a Receptor 1	down
*FIBIN*	1.384	0.0004	0.048	Fin Bud Initiation Factor Homolog	up
E) HIGH-HCHF vs NORM-CONV					
*ACHE*	−4.150	8.08E-05	0.033	Acetylcholinesterase (Cartwright Blood Group)	down
*ST14*	−3.840	0.0003	0.038	ST14 Transmembrane Serine Protease Matriptase	down
*PADI2*	−3.548	0.0002	0.036	Peptidyl Arginine Deiminase 2	down
*CRLF2*	−3.385	0.0005	0.044	Cytokine Receptor Like Factor 2	down
*HTRA4*	−3.358	0.0002	0.036	HtrA Serine Peptidase 4	down
*CD300E*	−3.210	0.0004	0.040	CD300e Molecule	down
*S100A5*	−3.211	0.0004	0.041	S100 Calcium Binding Protein A5	down
*NLRP1*	−3.117	4.80E-05	0.033	NLR Family Pyrin Domain Containing 1	down
*HK*3	−3.100	0.0001	0.033	Hexokinase 3	down
*ITGAX*	−3.084	0.0004	0.040	Integrin Subunit Alpha X	down
*FIBIN*	1.380	0.0004	0.040	Fin Bud Initiation Factor Homolog	up
*HMCN1*	1.269	0.0003	0.038	Hemicentin 1	up
*ETNPPL*	0.990	0.0006	0.047	Ethanolamine-Phosphate Phospho-Lyase	up
*ARHGAP20*	0.837	0.0006	0.047	Rho GTPase Activating Protein 20	up
*DHRS12*	0.780	0.0006	0.047	Dehydrogenase/Reductase 12	up
*LYRM1*	0.740	0.0006	0.047	LYR Motif Containing 1	up
*TRIM13*	0.593	0.0004	0.041	Tripartite Motif Containing 13	up
*DNAL1*	0.589	0.0005	0.042	Dynein Axonemal Light Chain 1	up
*PDRG1*	0.564	9.18E-05	0.033	P53 And DNA Damage Regulated 1	up
F) HCHF vs CONV					
*SPP1*	−4.788	5.45E-05	0.021	Secreted Phosphoprotein 1	down
*GPNMB*	−4.412	2.55E-05	0.019	Glycoprotein Nmb	down
*TREM2*	−3.924	3.08E-05	0.019	Triggering Receptor Expressed On Myeloid Cells 2	down
*SCIN*	− 3.612	2.75E-05	0.019	Scinderin	down
*LIPA*	−3.528	3.99E-05	0.020	Lipase A, Lysosomal Acid Type	down
*CAPG*	−3.374	4.07E-06	0.010	Capping Actin Protein, Gelsolin Like	down
*S100A4*	−3.350	2.19E-06	0.009	100 Calcium Binding Protein A4	down
*TYROBP*	−3.248	6.54E-05	0.021	Transmembrane Immune Signaling Adaptor TYROBP	down
*CTSZ*	−3.211	1.38E-05	0.019	Cathepsin Z	down
*MMP9*	−3.170	0.0006	0.034	Matrix Metallopeptidase 9	down
*LIPN*	5.163	3.63E-05	0.020	Lipase Family Member N	up
*DCST1*	4.950	0.0002	0.027	DC-STAMP Domain Containing 1	up
*TE*X11	4.896	1.88E-05	0.019	Testis Expressed 11	up
*ABCB5*	4.823	0.0003	0.028	ATP Binding Cassette Subfamily B Member 5	up
*CCDC148*	4.808	0.0001	0.023	Coiled-Coil Domain Containing 148	up
*FSIP2*	4.802	0.0003	0.028	Fibrous Sheath Interacting Protein 2	up
*C6orf58*	4.796	0.0004	0.030	Chromosome 6 Open Reading Frame 58	up
*MORC1*	4.780	0.0006	0.033	MORC Family CW-Type Zinc Finger 1	up
*DKK1*	4.776	6.43E-05	0.021	Dickkopf WNT Signaling Pathway Inhibitor 1	up
*CD28*	4.642	3.12E-06	0.010	CD28 Molecule	up

### Hub genes, top significant modules, and their respective enrichment identification via protein-protein interaction (PPI) network analyses of DEGs

**Fig. 2:**
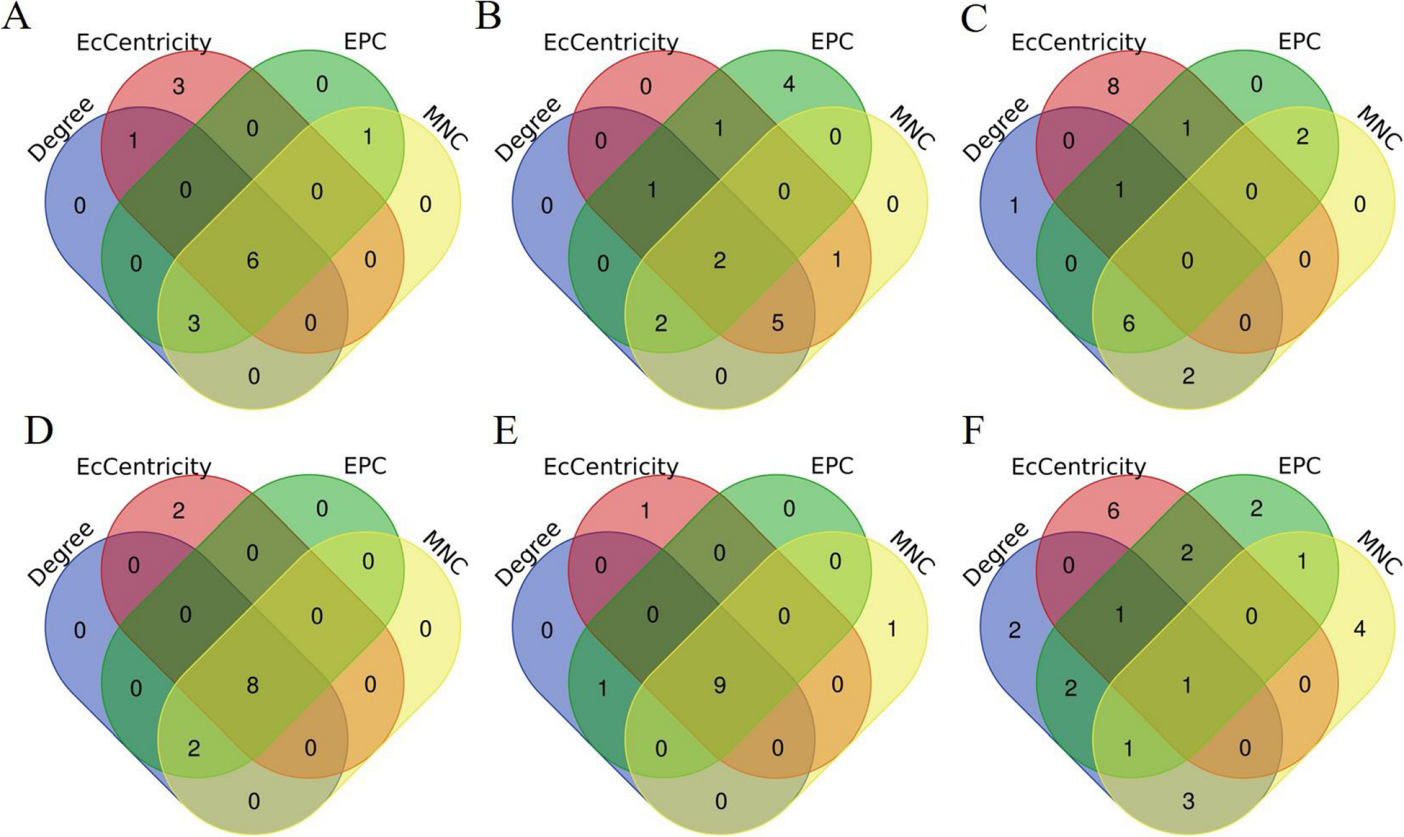
Venn diagram showing the number of top 10 ranked genes and hub genes. The hub genes were identified (genes overlapped in the four centrality methods) in the following comparisons: **a** LOW vs HIGH, **b** LOW vs NORM, **c** interaction between prenatal nutrition and sex (PreNxsex), **d** LOW-HCHF vs NORM-CONV, **e** HIGH-HCHF vs NORM-CONV, and **f** HCHF vs CONV. For the selection of hub genes, the PPI networks were first constructed using the DEGs with a high-confidence score < 0.70, followed by selection of top 10 ranked DEGs based on four centrality methods: Degree, EcCentrity, EPC, and MNC performed in the CytoHubba application (Cytoscape-plug in). Finally, genes that fell within all of these four criteria were considered as hub genes

The Cytoscape StringApp was used to visualize the long lists of DEG network. The DEG networks for all group comparison are shown in Additional file 2: Supplemetary Figure 1A-F. The top 10 DEGs evaluated in the PPI network according to four different centrality criteria (Degree, EcCentrity, EPC, and MNC) are shown in Table 3, and DEGs that topped the lists according to all four criteria were considered to be hub genes. Hence, a total of six hub genes for the LOW vs HIGH comparison, two hub genes for LOW vs NORM, eight hub genes for LOW-HCHF vs NORM-CONV, nine hub genes for HIGH-HCHF vs NORM-CONV, and one hub gene for HCHF vs CONV, were identified as shown in Fig. 2. Among all of the pairwise group comparison, no hub gene was identifed for the PreNxsex. The hubgenes identified for LOW vs HIGH were Aurora Kinase A (*AURKA*), Exonuclease 1 (*EXO1*), Maternal Embryonic Leucine Zipper Kinase (*MELK*) and PDZ Binding Kinase (PDK), NDC80 Kinetochore Complex Component (*NDC80* and *TTK Protein Kinase* (*TTK*). Those for LOW vs NORM were Coiled-Coil Domain Containing 39 (*CCDC39*) and Transkelotase Like 1 (*TKTL1*). The Complement C1q Chain (*C1QA*), Complement C1q B Chain (*C1QB*), Colony Stimulating Factor 1 Receptor (*CSF1R*), Cathepsin S (*CTSS*), Integrin Subunit Beta 2 (*ITGB2*) and Lysosomal Protein Transmembrane 5 (*LAPTM5*) were hub genes both for LOW-HCHF and HIGH-HCHF vs NORM-CONV group. Moreover, the Complement C5a Receptor 1 (*C5AR1*) and Protein Tyrosine Phosphatase Non-Receptor Type 6 (*PTPN6*) were hub genes for LOW-HCHF vs NORM-CONV, wheares Complement C1q C Chain (*C1QC*), Spi- Proto-Oncogene (*SP11*) and Transmembrane Immune Signaling Adaptor TYROBP (*TYROBP*) were hub genes for HIGH-HCHF vs NORM-CONV. The Matrix Metallopeptidase 9 (*MMP9*) was the only hub gene for HCHF vs CONV.

**Table 3:** The list of top 10 genes identified for six (A-F) group comparisons

Gene	Degree	Gene	EcCentricity	Gene	EPC	Gene	MNC
A) LOW vs HIGH							
***TTK***	15	***MELK***	0.009771	***TTK***	11.523	***TTK***	14
*AURKB*	14	***PBK***	0.009771	*MLPH*	11.41	*AURKB*	13
***NDC80***	13	*ESCO2*	0.009771	***NDC80***	11.369	***NDC80***	13
***AURKA***	13	***NDC80***	0.009771	***AURKA***	11.362	***AURKA***	13
*KIF2C*	12	***AURKA***	0.009771	*KIF2C*	11.282	*KIF2C*	12
***MELK***	11	***EXO1***	0.009771	***PBK***	11.094	***MELK***	11
***PBK***	11	*POLE*	0.009771	***MELK***	10.921	***PBK***	11
***EXO1***	10	***TTK***	0.009771	*CENPN*	10.474	*CENPN*	9
*CCDC39*	9	*CLSPN*	0.009771	*ASF1B*	9.639	***EXO1***	8
*CENPN*	9	*CCDC39*	0.007892	***EXO1***	9.508	*ASF1B*	7
B) LOW vs NORM							
***CCDC39***	**3**	***CCDC39***	0.024242	***CCDC39***	2.394	*NIPAL4*	1
***TKTL1***	2	***TKTL1***	0.018182	*RIMS2*	1.932	*RPH3A*	1
*RIMS2*	2	*RIMS2*	0.018182	***TKTL1***	1.923	***CCDC39***	1
*NIPAL4*	1	*NIPAL4*	0.012121	*HYDIN*	1.912	*HKDC1*	1
*RPH3A*	1	*RPH3A*	0.012121	*DNAH11*	1.899	*TTN*	1
*HKDC1*	1	*TTN*	0.012121	*ARMC4*	1.869	*HHIP*	1
*TTN*	1	*HHIP*	0.012121	*CNTNAP5*	1.696	*CNTNAP5*	1
*HHIP*	1	*ROBO3*	0.012121	*PCLO*	1.68	*ROBO3*	1
*CNTNAP5*	1	*USH2A*	0.012121	*TEX11*	1.679	*USH2A*	1
*ROBO3*	1	*ARMC4*	0.012121	*HKDC1*	1.666	***TKTL1***	1
C) CPreNxsex							
*WDFY1*	27	*BTAF1*	0.016998	*CDC5L*	38.608	*ENSOARG00000019688*	10
*ENSOARG00000019688*	11	*CDC5L*	0.014873	*ENSOARG00000010203*	35.73	*RSL24D1*	10
*NMD3*	10	*GTF2B*	0.014873	*RSL24D1*	34.388	*ENSOARG00000008494*	9
*PRKACB*	10	*CDKN2AIPNL*	0.013221	*ENSOARG00000019688*	34.338	*SRP54*	9
*SRP54*	10	*ENSOARG00000010203*	0.013221	*SRP54*	34.311	*ENSOARG00000001638*	9
*ENSOARG00000008494*	9	*TMPO*	0.013221	*ENSOARG00000008494*	34.285	*ENSOARG00000011629*	9
*ENSOARG00000001638*	9	*PRPF39*	0.013221	*ENSOARG00000016333*	34.274	*ENSOARG00000006781*	9
*ENSOARG00000011629*	9	*HNRNPC*	0.013221	*RPL37*	34.25	*LOC780467*	9
*ENSOARG00000006781*	9	*TCEA1*	0.013221	*ENSOARG00000001638*	34.249	*ENSOARG00000016333*	9
*LOC780467*	9	*MSH6*	0.013221	*LOC780467*	34.226	*RPL37*	8
D) LOW-HCHF vs NORM-CONV							
***ITGB2***	**11**	*CTSS*	0.062706	***ITGB2***	8.034	***ITGB2***	10
***CTSS***	9	***PTPN6***	0.062706	***C1QB***	7.933	***CTSS***	9
***C1QB***	9	***CSF1R***	0.062706	***CTSS***	7.817	***CSF1R***	8
***CSF1R***	8	***ITGB2***	0.062706	***CSF1R***	7.756	***C1QB***	8
*TYROBP*	8	***C5AR1***	0.04703	*TYROBP*	7.662	*SPI1*	8
*SPI1*	8	***C1QA***	0.04703	*SPI1*	7.568	***C1QA***	7
***C1QA***	7	*CYBB*	0.04703	***C1QA***	7.427	*TYROBP*	7
***LAPTM5***	7	***LAPTM5***	0.04703	***LAPTM5***	7.275	***LAPTM5***	6
***PTPN6***	4	*FN1*	0.04703	***PTPN6***	5.573	***C5AR1***	3
***C5AR1***	3	***C1QB***	0.04703	***C5AR1***	5.306	***PTPN6***	3
E) HIGH-HCHF vs NORM-CONV							
***ITGB2***	17	*C1QA*	0.038743	***ITGB2***	13.878	***ITGB2***	13
***TYROBP***	12	***LAPTM5***	0.038743	***C1QB***	13.461	***CTSS***	10
***CTSS***	11	***CTSS***	0.038743	***TYROBP***	13.449	***C1QB***	10
***C1QB***	10	***CSF1R***	0.038743	***CTSS***	13.19	***CSF1R***	9
***CSF1R***	9	***C1QC***	0.038743	***CSF1R***	13.163	***TYROBP***	9
***SPI1***	9	***C1QB***	0.038743	***C1QA***	12.963	***SPI1***	9
***C1QA***	8	***ITGB2***	0.038743	***SPI1***	12.862	***C1QA***	8
***LAPTM5***	8	*FCER1G*	0.038743	***C1QC***	12.328	***C1QC***	7
***C1QC***	7	***TYROBP***	*0.03874*	***LAPTM5***	*11.808*	***LAPTM5***	*6*
*PTPN6*	5	***SPI1***	*0.03874*	*PTPN6*	*9.368*	*C5AR1*	*3*
F) HCHF vs CONV							
*TYROBP*	10	***MMP9***	0.010243	*TYROB*	13.307	*ATP6V1B2*	6
*CXCR4*	8	*CD44*	0.010243	*ITGB*	12.993	*ATP6AP2*	5
*ATP6V1B*	6	*CXCR4*	0.008536	*CTS*	12.632	*ATP6V1C1*	5
*PTPN*	6	*TGFB1*	0.008536	*PTPN6*	12.551	*TCIRG1*	5
*CCDC39*	6	*CD28*	0.008536	*CXCR*	12.201	*ATP6V1E1*	5
*PKM*	6	*CYBB*	0.008536	***MMP9***	12.1	*ATP6V1A*	4
***MMP9***	6	*ALCAM*	0.008536	*LAPTM5*	11.938	*CTSS*	4
*ITGB2*	6	*ENSOARG0000000780*	0.008536	*CD44*	11.692	***MMP9***	4
*TCIRG1*	6	*SPP1*	0.008536	*ZAP70*	11.335	*ITGB2*	4
*ATP6V1A*	5	*ACTN2*	0.007682	*CYBB*	10.239	*ENTPD5*	3

The top 10 genes were identified according to four different criteria (Degree, EcCentricity, EPC and MNC) through the protein-protein interaction (PPI). Significant hub genes are highlighted in bold

**Fig. 3:**
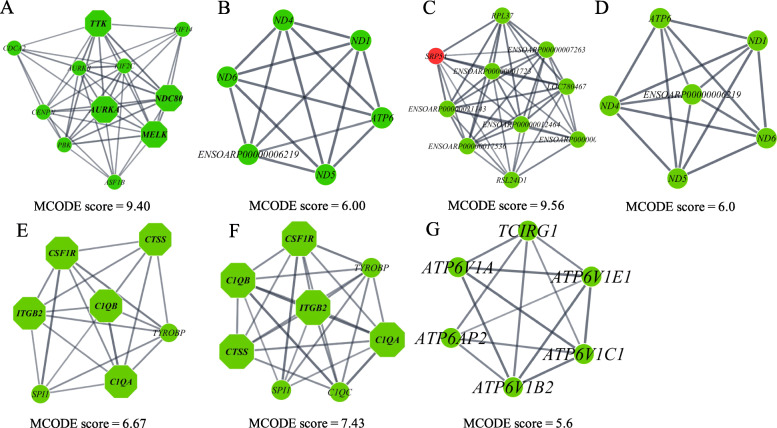
**a**-**g** The significant modules from PPI networks. **a**-**b** module 1 and module 2 for LOW vs HIGH, **c**-**d** module 1 and module 2 for interaction of prenatal nutrition and sex (PreNxsex), **e** module for LOW-HCHF vs NORM-CONV, **f** module for HIGH-HCHF vs NORM-CONV, and **g** module for HCHF vs CONV. The green and red nodes represent downregulated and upregulated genes, respectively, and the octagon nodes represent hub genes within the hub genes within the specific modules

Besides the selection of hub genes, we also identifed the top signifcant modules (sub-cluster) through the PPI networks analysis, of which modules having more than 6 nodes (genes) were selected. Two top modules were identified from the PPI network of the DEGs for LOW vs HIGH: module 1 with MCODE score = 9.40 with 11 nodes and 47 edges, and module 2 with MCODE score = 6.00 with 6 nodes and 15 edges. Two top significant modules were also observed for PreNxsex: module 1 with MCODE score = 9.56 with 10 nodes and 43 edges, and module 2 with MCODE score = 6.00 with 6 nodes and 15 edges. One significant module was identified for LOW-HCHF vs NORM-CONV (7 nodes and 20 edges), HIGH-HCHF vs NORM-CONV (8 nodes and 26 edges) and HCHF vs CONV (6 nodes and 14 edges) with MCODE scores of 6.67, 7.43 and 5.60, respectively, as shown in Fig. 3a-g. No significant modules were observed for LOW vs NORM.

To gain insight into the biological function of these modules, a functional enrichment analysis was performed with ClueGO. For LOW vs HIGH, module 1 was enriched in the group terms ‘attachment of mitotic spindle microtubules to kinetochore’ (74.47%), ‘mitotic sister chromatid segregation’ (21.28%), ‘mitotic nuclear division’ (2.13%) and ‘mitotic spindle organization’ (2.13%), whereas module 2 was enriched in ‘oxidoreductase activity, acting on NAD(P) H, quinone or similar compound as receptor’ (93.33%) and ‘oxidoreductase phosphorylation’ (6.67%). For the PreNxsex interaction, no functional enrichment was found for module 1, but similar to LOW vs HIGH, module 2 was enriched in ‘oxidoreductase activity, acting on NAD(P) H, quinone or similar compound as receptor’ (93.33%) and ‘oxidoreductase phosphorylation’ (6.67%). Both the LOW-HCHF and HIGH-HCHF vs NORM-CONV module was enriched in ‘myeloid leukocyte activation’ (100%). For HCHF vs CONV, the significant module was enriched in ‘collecting duct acid secretion’ (88.24%), ‘proton-transporting V-type ATPase complex’ (5.88%), and ‘proton-transporting two-sector ATPase complex’ (5.88%).

### Functional enrichment analyses of differentially expressed genes (DEGs)

#### DEGs of prenatal nutrition and prenatal x sex (PreNxsex)

The list of the most significant term of a group (leading term) for all comparison are shown in Table 4. Functional enrichment analyses revealed that GO terms, significantly enriched with genes differentially expressed between LOW vs HIGH and LOW vs NORM, were predominantly related to transmembrane (ion) transport activities, motor activities related to cytoskeletal and spermatozoa function (microtubules and the cytoskeletal motor protein, dynein), and responsiveness to the (micro) environmental extracellular conditions, including endocrine and nervous stimuli (Table 4). There were, however, specific terms, which distinguished LOW vs HIGH and not LOW vs NORM and vice versa. Thus, the term ‘positive regulation of vascular endothelial growth’ was enriched with DEGs between LOW vs HIGH, whereas the terms ‘homophilic adhesion via plasma molecules (cellular adhesion)’ and ‘lipid transporter activity’ were enriched with DEGs between LOW vs NORM.

**Table 4:** Significantly enriched biological functions of the DEGs

Group contrast	Ontology source	Biological function	Terms per group (%)
A) LOW vs HIGH	GO_Biological Process	transmembrane transport	14.23
	GO_Molecular Function	ATP binding	7.11
	GO_Biological Process	male gamete generation	6.3
	GO_Cellular Component	presynapse	5.89
	GO_Biological Process	ion channel activity	5.08
	GO_Biological Process	regulation of macromolecule metabolic process	6.67
	GO_Biological Process	ion transport	3.05
	GO_Cellular Component	sodium channel complex	2.89
		others	48.78
B) LOW vs NORM	GO_Biological Process	flagellated sperm motility	38.46
	GO_Biological Process	inorganic anion transmembrane transport	26.92
	GO_Cellular Component	postsynaptic density	7.69
		others	26.93
C) Interaction effect of prenatal nutrition and sex (PreNxsex)	GO_Cellular Component	dynein complex	21.43
	GO_Biological Process	inorganic molecular entity transmembrane transporter activity	20.00
	GO_Biological Process	cell development	8.57
	GO_Biological Process	ion transmembrane transporter activity	7.14
	GO_Molecular Function	motor activity	7.14
		others	35.72
D) LOW-HCHF vs NORM-CONV	GO_Biological Process	leukocyte activation	35.32
	GO_Biological Process	regulation of interleukin-6 production	10.04
	GO_Molecular Function	cell adhesion molecule binding	8.92
	GO_Biological Process	positive regulation of myeloid cell differentiation	7.81
	KEGG	Cholesterol metabolism	3.35
		others	34.56
E) HIGH-HCHF vs NORM-CONV	GO_Biological Process	leukocyte activation	18.43
	GO_Biological Process	regulation of immune system process	6.95
	GO_Biological Process	positive regulation of interleukin-1 production	4.02
	GO_Biological Process	regulation of leukocyte mediated immunity	3.15
	GO_Biological Process	positive regulation of leukocyte mediated immunity	3.07
		others	64.38
F) HCHF vs CONV			
Upregulated	GO_Biological Process	transmembrane transporter activity	26.27
	GO_Biological Process	regulation of postsynaptic membrane potential	9.91
	GO_Biological Process	regulation of natural killer cell mediated immunity	6.68
		others	57.14
Downregulated	GO_Biological Process	regulation of immune system process	15.35
	GO_Biological Process	inflammatory response	11.68
	GO_Cellular Component	proton-transporting V-type ATPase, V1 domain	3.00
	GO_Biological Process	actin cytoskeleton organization	3.84
	GO_Biological Process	cell differentiation involved in kidney development	0.25
		others	65.88

#### DEGs of postnatal nutrition and interaction of prenatal and postnatal nutrition

Most of the functional enrichment in relation to the early postnatal HCHF feeding were involved in immunity-related processes and pathways as well as transmembrane (ion) transport. Besides that, a biological process related to cell differentiation involved in kidney development was enriched among the downregulated DEGs in HCHF sheep.

In particular, 100 out of 101 and 180 out of 192 genes were downregulated in LOW-HCHF and HIGH-HCHF compared to NORM-CONV, respectively. The downregulated genes identified in both group contrasts (LOW-HCHF or HIGH-HCHF vs NORM-CONV) were associated with pathways involved in adipose tissue remodeling (stress response and apoptosis-related processes/pathways) and immunity-related processes/pathways. In addition, we found, the KEGG pathway related to ‘Cholesterol metabolism’ was enriched in LOW-HCHF compared to NORM-CONV, where the genes involved were downregulated in LOW-HCHF group.

## Discussion

In this study, we aimed to reveal the biological mechanisms and pathways involved in and/or responsible for tissue-specific (SUB and PER) responses to early life malnutrition, and to identify potential biomarkers (hub genes) by Next-Generation Sequencing transcriptomic analysis underlying these changes and their possible association to adverse metabolic and kidney developmental traits. We have previously demonstrated, in the sheep providing samples for this study, that SUB of adult sheep irrespective of their early life nutrition history, had similar upper limits for expandability, however with greater expandability capacity in females than males, whereas PER was a major target of early life nutritional programming, and a determinant for intra-abdominal fat distribution patterns [15]. Adult males that had been exposed to late gestation LOW level of nutrition followed by the mismatching HCHF diet in early postnatal life, had reduced hypertrophic capacity of PER, whereas fetally overnourished (HIGH) males apparently were resistant to this effect of the HCHF diet, and all HIGH sheep had increased PER hypertrophic expandability similar to what was observed in female sheep [15]. As previously mentioned, morphological features of SUB and PER were poorly correlated to changes in gene expression of well-known markers for adipose development, metabolism, angiogenesis and inflammation. Finally, the adult LOW-HCHF sheep, irrespective of sex, were hypercholesterolemic, hyperureaemic and hypercreatinaemic compared to all other groups, despite a preceding 2-years period of dietary correction after the exposure to the HCHF diet in early postnatal life [16, 17], and by the end of the exposure to the HCHF diet, the 6-months old HCHF lambs had massive deposition of fat in PER co-existing with a 1/3 reduction in kidney size [17].

In the present part of the study, we found gene expression profiles of PER, but not SUB were modulated by late gestation and early postnatal nutrition, and the latter even after the same low-fat hay-based diet had been fed to all sheep for 2 years. Irrespective of early life nutrition, only sex-specific differences in the expression of 44 mRNA were identified for SUB. Unsurprisingly, long-term effects of the pre-and postnatal nutrition were observed for mRNA expression of PER, especially as a consequence of a prenatal LOW level of nutrition. The majority (more than half) of DEGs identified in LOW were downregulated when compared to HIGH but in contrast opposite response was observed when compared to NORM (upregulated). The expression of DEGs between LOW/HIGH-HCHF and NORM-CONV were mostly downregulated, and only very few (less than 13) were upregulated in the former groups.

### The mRNA expression profiles of SUB in adult sheep were unaffected by the late gestation and early postnatal nutrition history

It is well-known that fat distribution patterns differ between males and females, with females accruing more fat in subcutaneous and gluteofemoral regions, whereas males have higher preference for lipid accumulation in the intra-abdominal area. It has been suggested that the greater susceptibility for central adiposity in males is linked to a higher predisposition for insulin resistance and cardiovascular diseases [10, 24, 25]. In contrast, both subcutaneous and gluteofemoral fat serves a protective role in this respect by preventing abdominal and ectopic fat (non-adipose tissues) depositions and associated adverse effects [7, 9].

We have recently shown in our sheep model of early life malnutrition that there was a marked reduction in intrinsic, non-obese cellularity in SUB in prenatally under- and overnourished (LOW and HIGH) adolescent lambs (6-months of age), but this difference was not evident in the adult sheep (2.5-years old). These findings suggest there must have been a time window for compensatory hyperplastic growth in this tissue, which was not related to the development of obesity [15, 26]. Moreover, in both lambs and adult sheep, and irrespective of the early life nutrition history, there was an upper-limit for hypertrophic expandability in subcutaneous adipocytes, with greater expansion capacity in females than males [15, 26], and this has also been observed in humans and murine model [13, 27, 28]. The lack of differences in mRNA expression in SUB of the adult sheep exposed to different combinations of nutrition in early life is consistent with these previous observations, and upper-limits for expandability in SUB do therefore not appear to be subject to late gestation programming. The present study could not contribute to shed light on the underlying mechanisms (e.g. molecular and pathways) that enabled sheep previously exposed to LOW or HIGH levels of nutrition in prenatal life to restore their hyperplastic ability from adolescence to adulthood. This warrants further studies. Nonetheless, as reported previously, the restoration of hyperplastic ability in SUB in prenatally malnourished sheep might be due to a generally reported higher adipogenic capacity [9, 29–31].

Our results, also support the so-called adipose expandability theory, stating that once the fat storing capacity of SUB is exhausted upon continuous excess nutrient supply, fat deposition will be directed towards the intra-abdominal region and ultimately towards non-adipocyte cell types, especially in males. SUB has thus been proposed as an initiating factor for the process of fat redistribution towards other anatomical sites, but not directly implicated in the development of obesity associated metabolic disturbances per se [27, 32–34]. As we have previously pointed out, the hitherto poorly studied PER thus becomes a major determinant for sex-specific intra-abdominal partitioning of fat deposition towards the mesenteric adipose tissue and ectopic regions [15].

### The biological functions and pathways underlying the long-term impacts of early nutrition on PER morphology and associated possible implications for metabolic disorders and kidney function

Very little scientific documentation exists on the role of PER (dys-)function in relation to obesity and related co-morbidities. Due to its anatomical location, PER has been studied mostly by non-invasive means in humans, and the easily accessible epididymal adipose tissue has been the main visceral adipose tissue studied in rodents. Nonetheless, the effect of feeding a high-protein neonatal formula to the suckling pigs on PER morphological traits [35] and feeding of different energy and nutrient sources for growing pigs on the transcriptome profiles of PER [36] has been previously documented. However, to our knowledge the impact of prenatal nutrition on PER has not been studied in pig model. We have previously reported that early obesity development induced by the HCHF diet in our sheep model had significant long-term implications for PER, since massive PER expansion and adipocyte hypertrophy in 6-months old lambs was associated with a collapse of its subsequent expandability later in life in contrast to 2 other adipose tissues studied (SUB and mesenteric) [16, 23]. It was therefore intriguing, that the present part of the study revealed major long-term impacts of the early nutrition history on expression of genes in PER. To the best of our knowledge, we are the first to demonstrate that nutrition in both late gestation and early postnatal life can alter gene expression profiles permanently in PER in adulthood, even after animals had been fed the exact same low-fat diet for the last 2 years of their lives. Our results demonstrate that prenatal malnutrition (especially LOW), in a long-term, can alter expression of genes participating in basic cell biology/structural functions and pathways related to transmembrane transport (e.g. ions, inorganic, and metal ions), motor activities involved in the cell cycle and sperm motility (e.g. microtubules, cilia, and the cytoskeletal motor protein dynein), and perception and response to various extracellular signals (e.g. macromolecule and growth factor stimuli). Similar to our findings, Leal and colleagues (2018) showed that enhanced nutrient supply during the pre-weaning period altered omental fat physiology of pre-weaned calves, and this was associated with an increase in omental fat mass (both hyperplasic and hypertrophic adipocyte expansion), altered biological function related to the cell cycle (e.g. mitosis and cell cycle progression), cellular assembly, organization (e.g. cytoskeleton formation) and molecular transport. Based on this evidence, it appears that PER is a target of prenatal nutrition, and PER as well as omental fat are targets of postnatal nutrition, and the long-term impacts may depend on the timing of the nutritional insult in relation to the biological window of their development [37–40].

Interestingly, early nutrition programing thus only appears to have long-term implications for some adipose tissues (perhaps depending on the timing of the intervention relative to functional development of the tissue). In PER, basic functions relating to cell structure, cytoskeletal motor function and processes involved in communicating information about the extracellular environment into the cell and cell nucleus were the targets of long-term programming, rather than metabolic processes per se. Various ion channels and transporters are involved in adipose proliferation and remodeling [41]. Metabolites such as glucose and amino acids, and ATP formation by mitochondrial activity are important facilitators for cell cycle progression [42], and these molecules are transported to the nucleus from the external environment or across mitochondrial membranes via various types of transmembrane transporters [43]. For example, ion channels have been shown to participate in the regulation of hormone release and adipocyte proliferation, of which the proliferating cells (compared to resting cells) exhibit ion channel expression. A number of ion channels have been identified in the pathogenesis of obesity [44]. Microtubule activities are crucial for the structural organization of cells, especially for maintenance of cell shape, division, motility, intracellular transport, regulation of cell polarity, modulation of cell adhesion and for control of force-production by the actin cytoskeleton [45, 46]. In addition, microtubules are one of the essential components involved in the cell cycle, required for the segregation of the complete set of chromosomes to each daughter cell [43]. Taken together, all of these pathways were altered especially in sheep with a history of prenatal LOW nutrition, demonstrating that early nutrition programming will interfere with the ability to develop and structure a normally functioning adipose tissue and its individual cells. However, to understand the implications of this in humans will require future research.

The pathological diseases related to abdominal and/or visceral adiposity/obesity has been extensively investigated in humans and animal models [14, 47, 48]. In this respect it was interesting that a pathway related to cholesterol metabolism was enriched in the DEGs identified between LOW-HCHF and NORM-CONV sheep, and expression of the involved genes were downregulated in LOW-HCHF sheep. Obesity is known to give rise to higher levels of circulating cholesterol in the blood, and this is a recognized factor in the development of coronary heart disease and also involved in regulation of adipocyte development (reviewed in [49]). The cholesterol content of adipose tissue is positively correlated to the size of adipocytes [50, 51]. Therefore, the persistent hypercholesterolemia observed in adult LOW (but not HIGH) sheep that had been exposed to the obesogenic HCHF diet in early postnatal life [16], could be explained by an impaired cholesterol metabolism associated with a reduced adipocyte hypertrophic ability in PER of especially LOW males [15] favoring lipid overflow into the mesenteric adipose and non-adipose tissues such as liver and blood [51].

### Early postnatal diet induced obesity altered the mRNAs expression of PER involved in cytokine and immunity-related pathways, partly depending on the prenatal nutrition history

Obesity is characterized by adipose tissue expansion due to increase in adipocyte size (hypertrophy) and/or numbers (hyperplasia), with visceral fat expansion as the primary target of early phase obesity development, followed by SUB, as shown using mouse models [9]. However, a sustained adipose expansion can lead to adipocyte death (apoptosis), tissue inflammation with formation of so-called crown-like structures, which ultimately results in adipose tissue dysfunction associated with various metabolic diseases [9]. Macrophages, eosinophils, T and B cells are among the immune cells responsible for the regulation of systemic as well as local immune homeostasis and inflammation, which consequently affect the adipose tissue and whole body metabolism [47, 52–54].

A maternal low-protein diet has been shown to impair development in the offspring of organs such as adipose tissues by redirecting the available nutrients to more critical organs such as the brain, and this will alter the ability of adipose tissues in postnatal life to adapt its metabolic function to a mismatching nutritional situation (e.g. a high fat diet) [48, 55–57].

In PER, we demonstrated significant alterations in the expression of mRNA playing a major role in adipose-related cytokine and immunity functions in both LOW and HIGH adult sheep previously exposed to the obesogenic HCHF diet, and the expression of most of the involved genes were downregulated compared to NORM-CONV sheep. These results corroborate a previous study in mice showing that maternal protein restriction during 2 weeks before mating and throughout gestation reduced the biological pathways activity related to cytokine production, innate immunity and phagocytosis in the male offspring fed a post-weaning high fat-diet. However, the authors also found increase in relative adiposity of the offspring, but this was not associated with adipose inflammation [48].

Intriguingly, the enrichment analysis of DEGs in PER, revealed that some of the DEGs were involved in biological functions related to kidney cell differentiation, and those genes were downregulated in the HCHF sheep compared to CONV sheep. In this respect, it is interesting that chronic kidney disease and compromised renal function has been associates with extreme PER expansion due to its direct functional (local toxic effects) and mechanical (compression) effects on the kidney [58–60] ascribed to their close proximity [61]. This may also contribute to the suggested regulatory role of PER on the cardiovascular system in addition to neural reflexes and adipokine secretion [62]. As previously mentioned, there was a 1/3 reduction of kidney weight in lambs from this trial that had been fed the HCHF as compared to CONV diet for 6 months [17], and this can be ascribed to the extreme PER fat mass observed in these lambs. However, there was apparently a ‘compensatory’ growth of the kidney from adolescence until adulthood in the HCHF sheep over the following 2 years period, when they were fed a low-fat hay-based [63]. Despite an apparently normalized kidney size, renal function may, therefore, have continued to be compromised after the early obesity development, and this could contribute to explain why the adult HCHF sheep were observed to be hypercreatinaemic and hyperureaemic compared to CONV sheep [16].

Our findings suggest that extreme PER expansion during early life can have persistent and adverse implications for PER susceptibility to inflammation in adulthood, particularly in individuals with a history of late gestation malnutrition (LOW or HIGH), in addition to potentially adverse implications for redistribution of fat deposition towards the mesenteric and ectopic regions as well as adult renal function.

### Hub genes that were affected by early life (mal) nutrition programming

The protein-protein interaction (PPI) network is comprised of highly connected protein nodes (known as hubs) and many poorly connected nodes. The deletion of a hub protein (gene) is thought to be more lethal than deletion of non-hub proteins [64]. This demonstrates the significance of hubs in organizing the network, which in turn suggests the biological importance of the network architecture, a key notion of systems biology [64]. Through RNA technologies (e.g. microarray and RNA-sequencing) and their respective bioinformatics downstream analyses, a vast number of hub genes (markers) related to adipose tissue and obesity co-morbidities have been identified [65–67].

In PER, we identified six hub genes known as *AURKA*, *EXO1*, *MELK*, *PDK*, *NDC80*, and *TTK*, whose expressions were downregulated in sheep with a history of a LOW as compared to HIGH level of nutrition in prenatal life. In addition, *CCDC39* and *TKTL1* were identified as upregulated hub genes between LOW vs prenatal NORM sheep. Among these hub genes, *AURKA*, *MELK*, and *TTK* have previously been shown to be expressed in adipose tissues, including in specific adipose-derived mesenchymal stem cells, and they are known to participate in biological functions and pathways related to the cell cycle, ciliary disassembly, adipogenesis, immunity, cell communication, cell motility, signal transduction and others [68–72]. Mesenchymal stem cells derived from adipose tissue of obese subjects exhibited shortened and deficient cilia, triggered by upregulation of ciliary disassembly regulators, like *AURKA*, resulting in a defect in adipogenesis, which in turn promoted adipocyte hypertrophy during the course of obesity [70]. In contrast, in vitro studies showed that adipose-derived mesenchymal stem cells cultured at low density (~ 50% confluent) had higher expression of proliferating-related genes, such as *AURKA* and *TTK*, whereas higher expression levels of immunity, cell communication, signal transduction and cell motility-related genes (e.g. cytokines, chemokines, and growth factors) were found in high-density cultures with 90% confluence [72]. It is possible that *AURKA* might play different roles depending on the state of obesity, and density of mesenchymal stem cells in adipose tissues.

In pig models, single nucleotide polymorphisms identified in the *CCDC39* gene have been shown to be associated with body fatness [73], whereas reduced expression of the *PDK* gene (involved in glucose transport regulation by insulin) were observed in insulin-resistance obese subjects. The *NCD80* gene was found to be expressed in stem cells derived from human exfoliated deciduous teeth [74]. Irregular expression of *NDC80* in cells is often accompanied with abnormal spindle checkpoint, abnormal chromosome separation and cell cycle disorders, and it has been found to lead to, and be highly expressed in, colon cancer cells [74, 75].

Based on these findings, we propose that adipocyte stem or precursor cells (involved in development and tissue repair) may be particular targets of long-term prenatal nutritional programming. The observations that spermatozoa, cilia and cytoskeletal motor functions were particular targets of prenatal malnutrition, suggests that fetal programming interfere with basal processes in the early assembly of (pre) adipocytes in PER. This can contribute to explain why prenatal malnutrition selectively targets certain tissues, depending on the timing of the insult relative to the specific time window of the fetal tissue formation and maturation. It can also contribute to explain why the prenatal programming is not readily apparent at birth, but only gradually becomes manifested as the individual ages and approaches adulthood.

The *ITGB2* and *CTSS* are hub genes with reduced expression in the PER of LOW-HCHF and HIGH-HCHF sheep as compared to NORM-CONV. *MMP9* was reduced in HCHF sheep compared to CONV sheep. The associations between these hub genes with obesity and obesity co-morbidities are well established. An increased expression of these hub genes were observed in SUB of obese Pima Indian subjects, and a single polymorphisms in the *ITGB2* were found in Japanese Americans, whose diet has become ‘Westernized’ compared to native Japanese [66, 76]. Other study showed that surgery-induced weight loss in morbidly obese women was associated with reduced CTSS content in SUB and circulating serum [77]. In agreement with study by Taleb and colleagues [77], we found that after a 2-years long period of dietary correction on a low-fat hay based diet, the expression of *CTSS* (but also *ITGB2*) were downregulated in the LOW-HCHF and HIGH-HCHF sheep, which were obese at the end of adolescence [23] compared to NORM-CONV.

## Conclusions

Our data revealed that the transcriptome of PER (but not SUB) was permanently altered by early life (mal) nutrition, with adverse implications observed particularly in adult sheep with a history of a LOW (but not HIGH) level of nutrition in late gestation upon exposure to a mismatching obesogenic diet in early postnatal life. It appears that in PER, fundamental genes (proteins) relating to biophysical sensing of the extracellular (micro) environment and early development/assembly of (pre) adipocytes are subject to fetal programming in response to late gestation LOW nutrition, which in turn might have implications for adipocytes proliferation, maturation and tissue organization. Some of the hub genes identified were *AURKA*, *MELK*, and *TKT* for prenatal LOW vs HIGH nutrition, and *CTSS* and *ITGB2* for late gestation malnutrition followed by early obesity development (LOW-HCHF and HIGH-HCHF vs NORM-CONV). *MMP9* was the only hub genes detected to be independently affected by the early postnatal diet in PER from adult sheep. Intriguingly, the hub genes, whose expressions were altered by prenatal nutrition, have previously been shown to be expressed in adipose-derived mesenchymal stem cells and participate in biological functions crucial for adipose tissue remodeling, which could explain why programming effects become manifested only, if the programming occurs in specific windows of PER development. Affected pathways in PER furthermore included cholesterol metabolism in LOW-HCHF sheep and impaired kidney cell differentiation across all HCHF sheep, which could contribute to explain the previously reported observations of persistent hypercholesterolemia, hyperuricemia, and hypercreatinemia in the nutritionally mismatched LOW-HCHF sheep.

## Methods

### Ethic statement

Animal experiments were conducted at an experimental barn at Rosenlund, Lynge, Denmark under the inspection of the Faculty of Health and Medical Sciences, University of Copenhagen. All the experimental procedures were approved by the National Committee on Animal Experimentation, Denmark (License no: 2010/561–1853).

### Experimental design, animals, diets and tissue collection

**Fig. 4:**
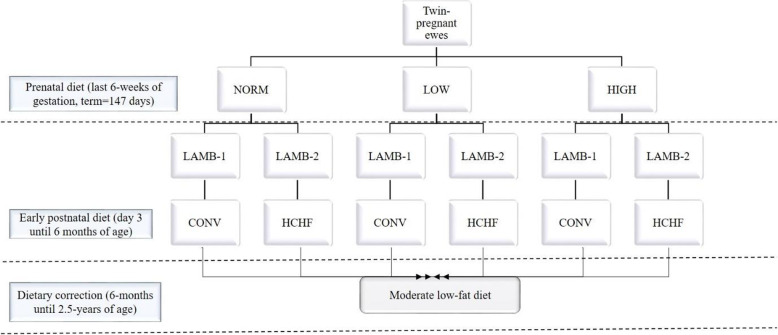
The flow chart of the experimental design and treatment groups. Prenatal diet (the last 6 weeks of gestation, terms = 147 days): NORM (fulfilling 100% of daily energy and protein requirements), LOW (providing 50% of NORM), or HIGH (providing 150% of daily energy and 110% of daily protein requirements). The early postnatal diet (day 3 until 6 months of age): CONV (moderate low fat diet consisting of hay supplemented during the first 8 weeks of life with milk replacer; amounts were adjusted to ensure moderate growth rates of appr. 225 g/d) and HCHF (high-carbohydrate (starch)-high-fat diet consisting of a mix of 37% fat dairy cream with milk replacer in a 1:1 ratio (max. 2½ L/d) supplemented with rolled maize (max. 2 kg/d) and barley straw). The experimental design thus gave rise to six treatment groups: NORM-CONV (*N* = 9, 5 males:4 females), and NORM-HCHF (*N* = 9, 6 males:3 females), LOW-CONV (*N* = 13, 6 males:7 females), LOW-HCHF (*N* = 13, 5 males:8 females), HIGH-CONV (*N* = 13, 5 males:8 females), HIGH-HCHF (*N* = 13, 5 males:8 females)

The experimental design (animals, diets and management) has been described elsewhere [16, 17]. In brief, the experiment was a 3 × 2 factorial design, in which a total of 36 twin-pregnant crossbred Texel ewes during the last 6 weeks of gestation (term = 147 days) were allocated to three different diets: NORM (fulfilling energy and protein requirements according to Danish feeding standards), LOW (50% of NORM) or HIGH (150 and 110% of daily energy and protein requirements, respectively). The lambs were separated from their dams 3 days after birth and twin lambs were subjected to each of their assigned diets until 6-months of age (after puberty): a moderate conventional diet, CONV (hay supplemented for the first 8 weeks of life with milk replacer; adjusted to achieve moderate and constant growth rates of approximately 225 g day^− 1^) or a high-carbohydrate-high-fat diet, HCHF (mix of 37% fat dairy cream with milk replacer in a 1:1 ratio (max. 2½ L/day) supplemented with rolled maize (max. 2 kg/day) and a small amount of barley straw) (Fig. 4). Subgroups of lambs from each of the 6 treatment groups were slaughtered at 6-months of age, while remaining animals were managed in two sex-divided groups and fed the same low-fat hay-based diet ad libitum until 2.5-years of age (supplemented with barley until 1.5-years of age). All animals had ad libitum access to water and a vitamin-mineral mix at all times. The experimental animals were slaughtered as 2.5-years old adult sheep by exsanguination, while under general anesthesia induced intravenously with Propofol (B.Braun, Melsungen, Germany; 5–6 mg kg^− 1^ body weight). At autopsy, specimens of subcutaneous (SUB; above the *M. longissimus dorsi*) and PER were sampled and immediately collected in RNA*later* (RNA*later*@Solution, Ambion, The RNA Company, USA) for 24 h. Finally, all samples were transferred into empty tubes and stored at − 80 °C pending analysis.

### RNA isolation, cDNA library construction and sequencing

Total RNA extraction, cDNA library construction and sequencing was performed by Novogene (HK) Company Limited, Hong Kong. The total RNA extraction method used was a Trizol protocol. The quality control of total RNA was performed using three methods as followed; 1) total RNA was preliminarily quantified in Nanodrop (Thermo Fisher Scientific, Carlsbad, CA, USA); 2) RNA degradation and potential contamination was tested by Agarose Gel Electrophoresis; and 3) the quantity and integrity of total RNA was checked using an Agilent 2100 Bioanalyzer (Agilent Technologies, Santa Clara, CA, USA), and RNA concentration was measured using the Qubit® RNA Assay Kit and a Qubit® 2.0 Fluorometer (Life Technologies, Carlsbad, CA, USA).

After quality control of RNA, ribosomal RNAs (rRNAs) were removed by Ribo-Zero™ rRNA Removal Kit (EPICENTRE® Biotechnologies, Madison, Wisconsin, USA). The purified RNA was first fragmented randomly to short fragments of 250 and 300 based-pair in fragmentation buffer, and then the first-strand cDNA was synthesized by adding random hexamer primers. After that, a custom second-strand synthesis buffer (Illumina, USA), dNTPs (dUTP, dATP, dGTP and dCTP) and DNA polymerase I were added to synthesize the second-strand. The resulting double-stranded DNA was purified by AMpure XP beads, and a poly A tail was ligated to the sequencing joint. The correct-sized fragments were purified by AMPure XP beads. The USER Enzyme (USER® Enzyme, New England, BioLabs® Inc., UK) was used to degrade the cDNA strands containing U instead of T, and the first strand cDNA was sequenced, thereby preserving the direction of the RNA. Finally, PCR amplification was conducted and the products were purified (AMPure XP beads) for constructing the cDNA libraries. The quality of the latter was assessed using Agilent BioAnalyzer 2100 system and qPCR. The libraries were sequenced on an Illumina Hiseq 4000 platform, and 150 long paired-end reads were generated.

### RNA-Seq and statistical analysis

**Fig. 5:**
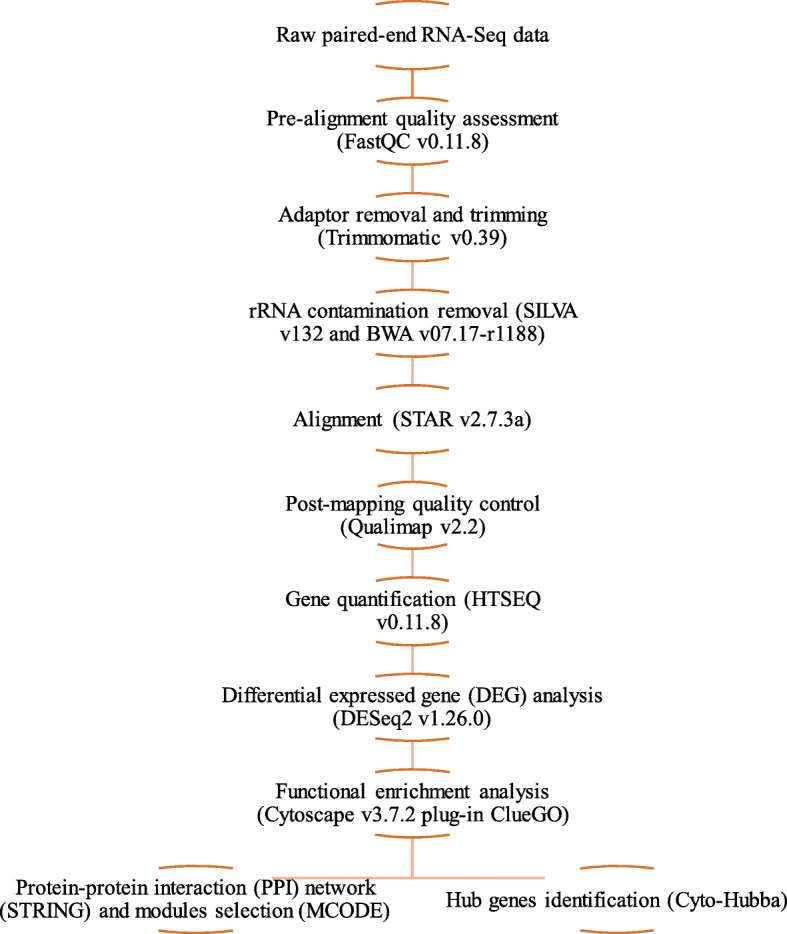
Overview of the work pipeline of differential expressed genes analysis

The bioinformatic pipeline for identification of differentially expressed genes (DEGs) is depicted in Fig. 5. The sequence quality or paired-end reads of each sample were assessed using FastQC v0.11.8 [78]. The result showed high quality of the raw sequences, but different levels of rRNA sequences. The Trimmomatic v0.39 [79] was used to remove potential adapter sequences and trimming of low quality reads. To achieve optimal coverage, good detection sensitivity, and reliable results, screening and removal of rRNA contamination of the paired-end reads were done using the database from SILVA v132 [80]. The rRNA sequences were retried from SILVA, and the Burrows-Wheeler-Aligner (BWA) v0.7.17-r1188 software [81] was used to build the index file for BWA. The BWA-MEM was used to map to the clean data to the SILVA database, and then the paired-end unmapped sequences were extracted using samtools v1.10 [82] and bedtools v2.29.2 [83]. The generated ‘clean reads’ were used for all the subsequent downstream bioinformatics analyses. The paired-end clean reads of each sample were mapped to the reference genome Oar v3.1.99 gtf [84] using STAR v2.7.3a [85] with a maximum of 8 mismatches allowed, and others parameters were set as default. A post-alignment quality control was performed on the alignment files using Qualimap v2.2 [86]. After mapping, each sequence alignment map (SAM) file obtained was sorted and transformed into a binary alignment map (BAM) file by Samtools v1.9 [82], and used for the gene counting. The gene expression counts were computed using HTSeq vo.11.8 [87]. The low count genes were filtered by excluding less than 1 count per million (cpm) and the DEGs were identified using DESeq2 v1.26.0 package [88] in RStudio (v1.2.5042) [89]. The DEGs were considered at a False Discovery Rate (FDR) < 0.05. The gene counts were normalized using the default normalization procedures provided by DESeq2 (correcting for the library size and RNA composition biases) and fitted with the following model:

$$ Y= sex+ PostN+ PreN+ sex: PostN+ sex: PreN+ PostN: PreN $$

Where *Y* is the gene expression count, *PreN* is the fixed effect of prenatal nutrition (NORM, LOW and HIGH), *PostN* is the fixed effect of the early postnatal nutrition (CONV and HCHF), and sex is the fixed effect of sex (♂ = males and ♀ = females). In the analyses, we considered the two-way interaction terms of sex*: PostN* (2 PostN × 2 sex), *sex: PreN* (3 prenatal nutrition × 2 sex) and *PostN: PreN* (3 prenatal nutrition × 2 early postnatal nutrition).

### Top modules selection and hub genes identification via protein-protein interaction (PPI) network

To select the top significant modules and identify the hub genes, we followed the method described by Yang et al. [90]. First, the PPI network was constructed to evaluate the DEGs relationship with a high-confidence score > 0.7 defined as significant using the Cytoscape StringApp [91]. Secondly, screening of sub-clusters (modules) of PPI networks was performed through the Molecular Complex Detection (MCODE) [92] application in Cytoscape. The MCODE criteria were set default with degree of cutoff = 2, node score cutoff = 0.2, and k-core, maximum depth = 100. The modules with more than 6 nodes (genes) were selected as top modules followed by the functional enrichment analysis of these modules using Cytoscape v3.7.2 plug-in ClueGO [91, 93]. The hub genes were identified using the Cyto-Hubba application in Cytoscape through four centrality methods, including Degree, Edge Percolated Component (EPC), Eccentricity, and Maximum Neighborhood Component (MNC) [94] with the cutoff criterion of > 10 top DEGs for further selection of hub genes [90]. The top 10 DEGs, which fell within the intersection of these four algorithm methods, were visualized in a Venn plot (http://bioinformatics.psb.ugent.be/webtools/Venn/), and were selected as hub genes.

### Functional enrichment analysis

The functional enrichment analysis was performed to further explore the biological functions and pathways associated with the significant DEGs. Each DEG set identified with padj < 0.05 were grouped into two sets of downregulated or upregulated genes, and were imported into Cytoscape v3.7.2 plug-in ClueGO [93, 95] to find significant enrichments using *Ovis aries* (Taxonomy ID: 9940) as the reference organism. The selection criteria for Gene Ontology (GO) terms (Biological Process, Cellular Component and Molecular Process) and Kyoto Encyclopedia of Genes and Genomes (KEGG) pathways were based on two-sided hypergeometric tests with *P*-value threshold < 0.05, which was corrected for multiple testing using the Benjamini-Hochberg false discovery rate (padj). Thresholds of a minimum of 3–20 GO levels, and a minimum number of 3 genes, or at least 4% genes in the respective terms, were applied.

## Supplementary Information

**Additional file 1: Table S1.** The list of differential expressed genes for males versus females in subcutaneous adipose tissue.

**Additional file 2: Figure S1 A-F.** The protein-protein interaction (PPI) networks of differentially expressed genes (DEGs). A) LOW vs HIGH, B) LOW vs NORM, C) interaction effect of prenatal nutrition and sex (PreNxsex), D) LOW-HCHF vs NORM-CONV, E) HIGH-HCHF vs NORM-CONV, and F) HCHF vs CONV. The red and green nodes represent upregulated and downregulated genes, respectively. The nodes with octagon shape are the hub genes. **Figure S2A-E:** Functional enrichment networks. A and B) module 1 and module 2 for LOW vs HIGH, C) module 2 for the interaction of prenatal nutrition and sex, D) module 1 for LOW-HCHF and HIGH-HCHF vs NORM-CONV and E) module 1 for HCHF vs CONV.

## Data Availability

The data presented in this work is available within this paper and its Additional files 1 and 2. The RNA-Seq data analyzed, discussed and presented in this study was deposited in NCBI’s Gene Expression Omnibus (GEO) and are accessible through GEO series accession number GSE166662 in the following link: https://www.ncbi.nlm.nih.gov/geo/query/acc.cgi?acc=GSE166662.

## Abbreviations

bp: Based-paired
BAM: Binary Alignment Map
CFP: Central for Foetal Programming
cDNA: Complementary DNA
CONV: Conventional low fat hay-based diet
DEGs: Differential Expressed Genes
EPC: Edge Percolated Component
FC: Fold Change
GO: Gene Ontology
HCHF: High-Fat-High-Carbohydrate
PreNxsex: Interactions of prenatal nutrition and sex
KEGG: Kyoto Encyclopedia of Genes and Genome
log2FC: Log2 Fold Change
MNC: Maximum Neighborhood Component
mRNA: Messenger RNA
MCODE: Molecular Complex Detection
NORM: Normal nutrition
HIGH: Overnutrition
PER: Perirenal adipose tissue
PCR: Polymerase Chain Reaction
PostN: Postnatal nutrition
PreN: Prenatal nutrition
PPI: Protein-Protein Interaction
qPCR: Quantitative Real-Time Polymerase Chain Reaction
RNA: Ribonucleic Acid
rRNA: Ribosomal RNA
SAM: Sequence Alignment Map
SUB: Subcutaneous adipose tissue
LOW: Undernutrition
