# Supplementary material for: Transcriptomics analysis of differentially expressed genes in subcutaneous and perirenal adipose tissue of sheep as affected by their pre- and early postnatal malnutrition histories
Source: BMC Genomics. 2021 May 11;22:338. doi: 10.1186/s12864-021-07672-5 (PMC8114714; doi:10.1186/s12864-021-07672-5)
Supplement: Supplementary file 1 — Additional file 1: Table S1. The list of differential expressed genes for males versus females in subcutaneous adipose tissue. [file 12864_2021_7672_MOESM1_ESM.docx]

**Additional File 1**

**Transcriptomics analysis of differentially expressed genes in subcutaneous and perirenal adipose tissue of sheep as affected by their pre- and early postnatal malnutrition histories**

*Sharmila Ahmad^1^, Markus Hodal Drag^2^, Suraya Mohamad Salleh^3,4^, Zexi Cai^5^, Mette Olaf Nielsen^1^*

*^1^Nutrition Research Unit, Department of Animal Science, Aarhus University, Blichers Alle 20, 8830 Tjele, Denmark,* [*sharmila_ahmad@anis.au.dk*](mailto:sharmila_ahmad@anis.au.dk)*, ^2^Novo Nordisk Foundation Center for Basic Metabolic Research, Faculty of Health and Medical Sciences, University of Copenhagen, Blegdamsvej 3B, 2200, Copenhagen, Denmark,* [*markus.drag@sund.ku.dk*](mailto:markus.drag@sund.ku.dk)*, ^3^Department of Animal Science, Universiti Putra Malaysia, 43400 Serdang, Selangor, Malaysia,^4^ Department of Animal Nutrition and Management, Swedish University of Agricultural Sciences, 750 07 Uppsala, Sweden,* [*surayams@upm.edu.my*](mailto:surayams@upm.edu.my)*, ^5^Centre for Quantitative Genetics and Genomics, Aarhus University, Blichers Alle 20, 8830 Tjele, Denmark,* [*zexi.cai@qgg.au.dk*](mailto:zexi.cai@qgg.au.dk)

*Corresponding author: *mon@anis.au.dk*

*^1^Nutrition Research Unit, Department of Animal Science, Aarhus University, Blichers Alle 20, 8830 Tjele, Denmark*

**Supplementary Table 1**: The list of differential expressed genes for males *versus* females in subcutaneous adipose tissue

| Gene.stable.ID | Gene.name | baseMean | log2FoldChange | padj |
| --- | --- | --- | --- | --- |
| ENSOARG00000000033 | *ND6* | 6078.071 | 6.258209 | 0.027285 |
| ENSOARG00000000522 | *SLITRK5* | 28.28822 | 4.145156 | 0.001657 |
| ENSOARG00000001851 | *FGF1* | 212.0032 | -4.65553 | 1.07E-06 |
| ENSOARG00000002554 | *SLC22A18* | 13.19157 | 5.5739 | 0.027285 |
| ENSOARG00000002686 |  | 29.727 | 6.46322 | 0.02521 |
| ENSOARG00000003928 | *HNF4A* | 432.0921 | -3.44773 | 0.00946 |
| ENSOARG00000003997 | *HYDIN* | 60.71845 | 4.945196 | 0.033548 |
| ENSOARG00000004606 | *COL22A1* | 125.4402 | 7.747001 | 0.004289 |
| ENSOARG00000006640 | *BLVRB* | 166.5924 | -1.51217 | 0.047825 |
| ENSOARG00000007230 | *ERICH4* | 175.1323 | 3.053738 | 0.007278 |
| ENSOARG00000007716 | *BPGM* | 49.32569 | -1.40693 | 0.033499 |
| ENSOARG00000007972 | *HLA-DMA* | 51.64006 | -1.42158 | 0.033499 |
| ENSOARG00000008011 | *GALNT13* | 58.55039 | 3.16243 | 0.006331 |
| ENSOARG00000008438 | *GM2A* | 977.0592 | -2.78927 | 0.007278 |
| ENSOARG00000008959 | *SLIT1* | 27.96096 | 5.405722 | 0.049747 |
| ENSOARG00000009032 | *SPARC* | 35580.74 | -2.7419 | 0.001657 |
| ENSOARG00000009230 | *DPT* | 2242.436 | -4.06788 | 0.005548 |
| ENSOARG00000009915 | *SULT1B1* | 28.03969 | 2.732954 | 0.033548 |
| ENSOARG00000010025 |  | 16.42687 | 5.99071 | 0.006331 |
| ENSOARG00000010290 | *PLXDC1* | 113.5075 | -2.36653 | 0.04215 |
| ENSOARG00000010746 | *PMEL* | 211.5403 | 3.582009 | 0.003488 |
| ENSOARG00000011377 |  | 102.8097 | -4.92841 | 0.007698 |
| ENSOARG00000011846 | *NTN4* | 540.706 | -1.46541 | 0.027285 |
| ENSOARG00000012275 |  | 35.6982 | 5.545445 | 0.029135 |
| ENSOARG00000012693 | *GPR153* | 441.9983 | -0.82796 | 0.029268 |
| ENSOARG00000012717 | *CLGN* | 20.90184 | 4.505353 | 0.027285 |
| ENSOARG00000013535 | *ITIH5* | 4433.495 | -2.09821 | 0.02521 |
| ENSOARG00000013777 |  | 415.7809 | 2.301597 | 0.019652 |
| ENSOARG00000014454 |  | 120.1846 | 7.6161 | 0.003488 |
| ENSOARG00000014685 | *SLC2A12* | 124.0954 | -2.73352 | 0.007144 |
| ENSOARG00000015051 | *BCAS1* | 15.33621 | 5.619109 | 0.027285 |
| ENSOARG00000015486 | *AXIN2* | 35.90607 | 3.927792 | 0.047764 |
| ENSOARG00000016144 | *LPIN1* | 637.0556 | 1.697213 | 0.024472 |
| ENSOARG00000016976 | *VGLL3* | 220.6078 | -2.4618 | 0.047825 |
| ENSOARG00000017299 | *TDRD5* | 28.98612 | 5.582832 | 0.012771 |
| ENSOARG00000017769 | *CITED1* | 19.03177 | 4.809505 | 0.001657 |
| ENSOARG00000018232 |  | 1706.415 | -3.94035 | 9.16E-06 |
| ENSOARG00000018988 | *KCNK3* | 29.83317 | 3.937224 | 0.027285 |
| ENSOARG00000019022 |  | 217.7061 | 2.21208 | 0.045743 |
| ENSOARG00000019246 | *PNPLA3* | 202.6304 | 3.65856 | 0.027285 |
| ENSOARG00000019716 | *CYP27A1* | 539.157 | -1.95317 | 0.041117 |
| ENSOARG00000021154 | *SPTB* | 91.49757 | 3.270479 | 0.024472 |
| ENSOARG00000026307 |  | 61.85223 | 8.049557 | 0.01262 |
| ENSOARG00000026594 |  | 18.80267 | 5.911428 | 0.047825 |

The positive and negative values of log2FoldChange represent up- and downregulated expression levels of the genes in males compared to females (reference groups). The DEGs were considered at a False Discovery Rate (FDR) <0.05.
