# Supplementary material for: Transcriptomics analysis of differentially expressed genes in subcutaneous and perirenal adipose tissue of sheep as affected by their pre- and early postnatal malnutrition histories
Source: BMC Genomics. 2021 May 11;22:338. doi: 10.1186/s12864-021-07672-5 (PMC8114714; doi:10.1186/s12864-021-07672-5)
Supplement: Supplementary file 2 — Additional file 2: Figure S1 A-F. The protein-protein interaction (PPI) networks of differentially expressed genes (DEGs). A) LOW vs HIGH, B) LOW vs NORM, C) interaction effect of prenatal nutrition and sex (PreNxsex), D) LOW-HCHF vs NORM-CONV, E) HIGH-HCHF vs NORM-CONV, and F) HCHF vs CONV. The red and green nodes represent upregulated and downregulated genes, respectively. The nodes with octagon shape are the hub genes. Figure S2A-E: Functional enrichment networks. A and B) module 1 and module 2 for LOW vs HIGH, C) module 2 for the interaction of prenatal nutrition and sex, D) module 1 for LOW-HCHF and HIGH-HCHF vs NORM-CONV and E) module 1 for HCHF vs CONV. [file 12864_2021_7672_MOESM2_ESM.docx]

**Additional File 2**

**Transcriptomics analysis of differentially expressed genes in subcutaneous and perirenal adipose tissue of sheep as affected by their pre- and early postnatal malnutrition histories**

*Sharmila Ahmad^1^, Markus Hodal Drag^2^, Suraya Mohamad Salleh^3,4^, Zexi Cai^5^, Mette Olaf Nielsen^1^*

*^1^Nutrition Research Unit, Department of Animal Science, Aarhus University, Blichers Alle 20, 8830 Tjele, Denmark,* [*sharmila_ahmad@anis.au.dk*](mailto:sharmila_ahmad@anis.au.dk)*, ^2^Novo Nordisk Foundation Center for Basic Metabolic Research, Faculty of Health and Medical Sciences, University of Copenhagen, Blegdamsvej 3B, 2200, Copenhagen, Denmark,* [*markus.drag@sund.ku.dk*](mailto:markus.drag@sund.ku.dk)*, ^3^Department of Animal Science, Universiti Putra Malaysia, 43400 Serdang, Selangor, Malaysia,^4^ Department of Animal Nutrition and Management, Swedish University of Agricultural Sciences, 750 07 Uppsala, Sweden,* [*surayams@upm.edu.my*](mailto:surayams@upm.edu.my)*, ^5^Centre for Quantitative Genetics and Genomics, Aarhus University, Blichers Alle 20, 8830 Tjele, Denmark,* [*zexi.cai@qgg.au.dk*](mailto:zexi.cai@qgg.au.dk)

*Corresponding author: *mon@anis.au.dk*

*^1^Nutrition Research Unit, Department of Animal Science, Aarhus University, Blichers Alle 20, 8830 Tjele, Denmark*


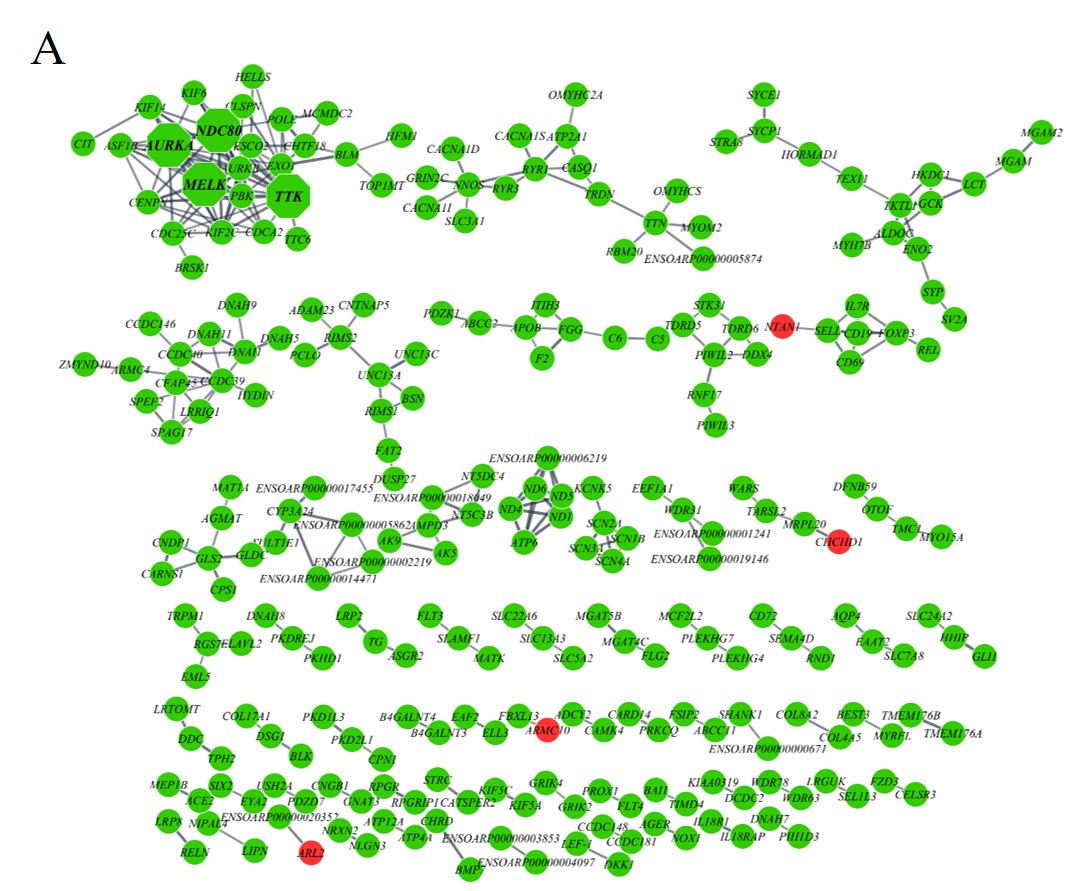


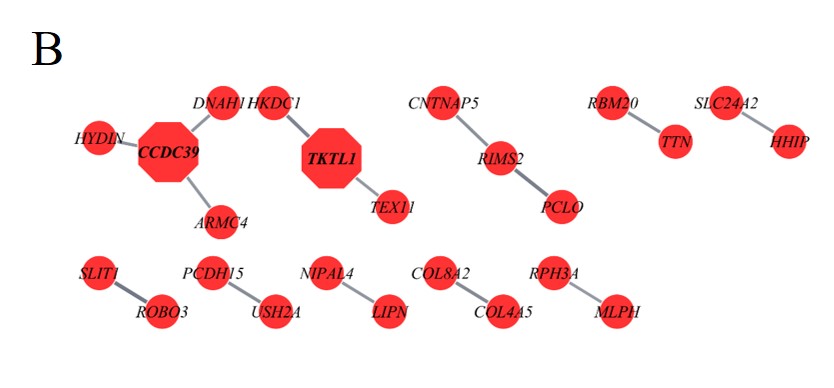


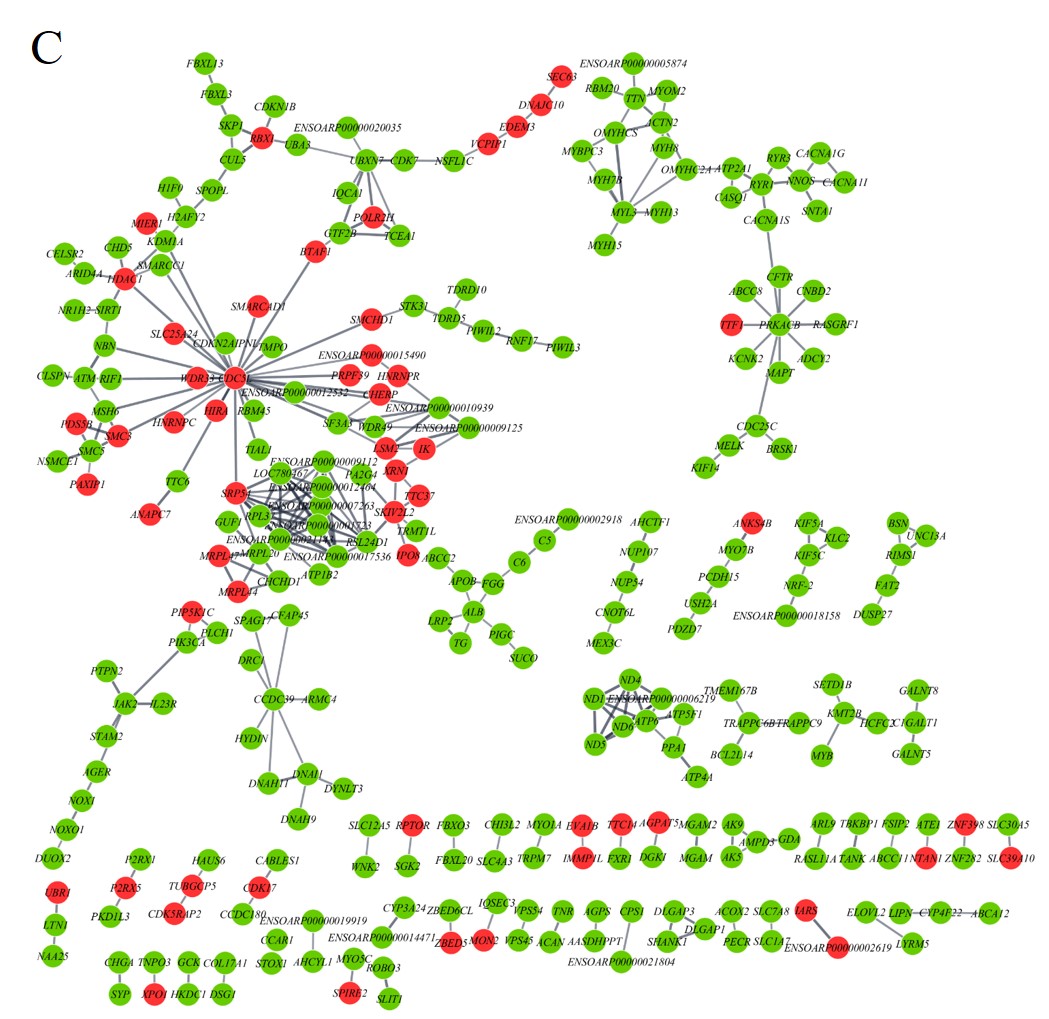


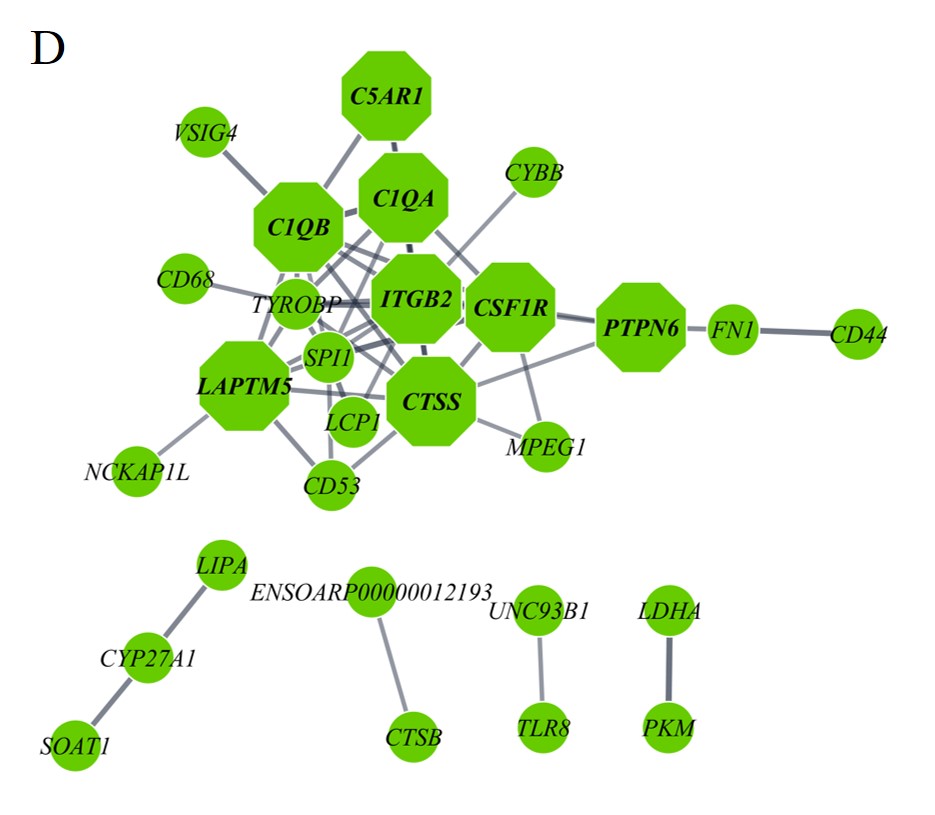


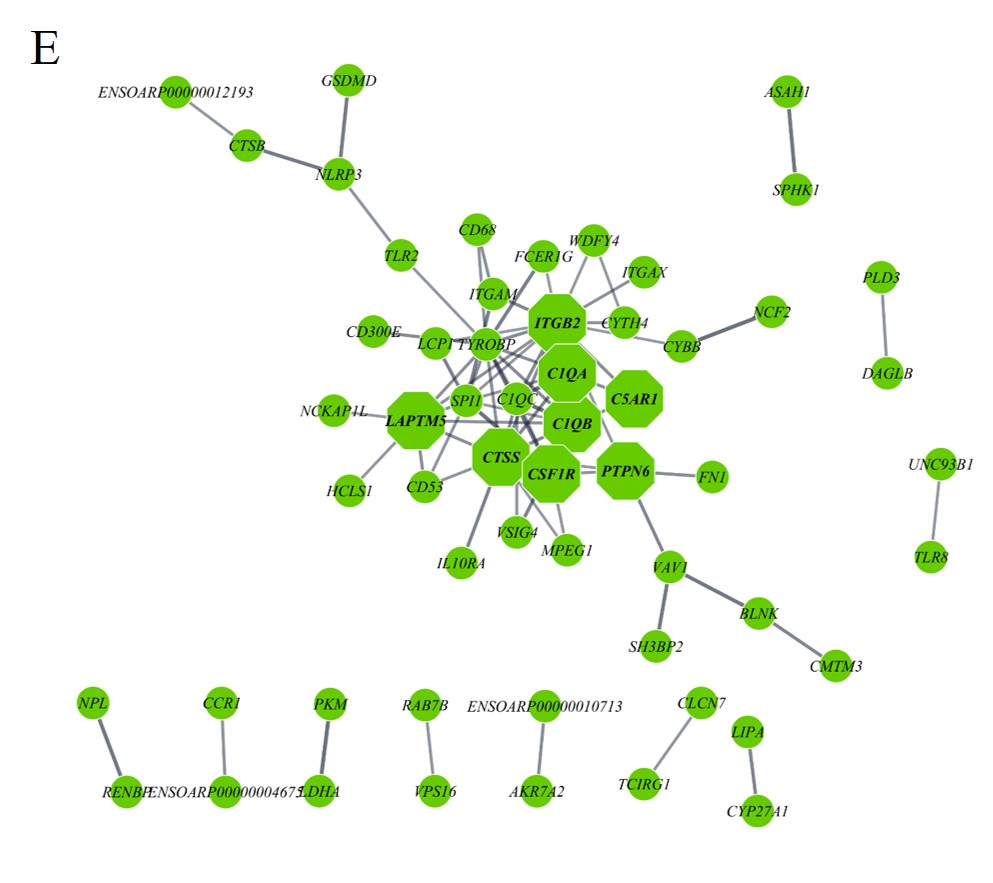


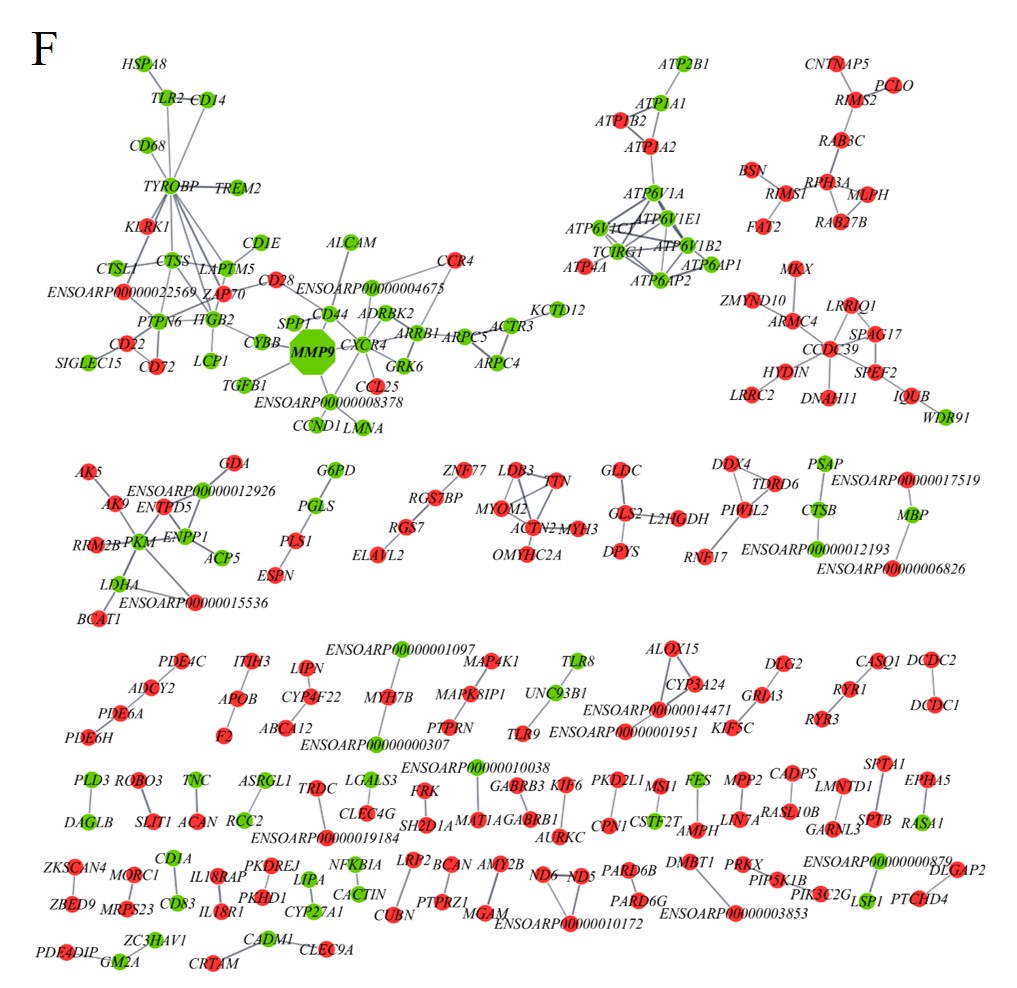


Supplementary Figure 1 A-F: The protein-protein interaction (PPI) networks of differentially expressed genes (DEGs). A) LOW *vs* HIGH, B) LOW *vs* NORM, C) interaction effect of prenatal nutrition and sex (PreNxsex), D) LOW-HCHF *vs* NORM-CONV, E) HIGH-HCHF *vs* NORM-CONV, and F) HCHF *vs* CONV. The red and green nodes represent upregulated and downregulated genes, respectively. The nodes with octagon shape are the hub genes.


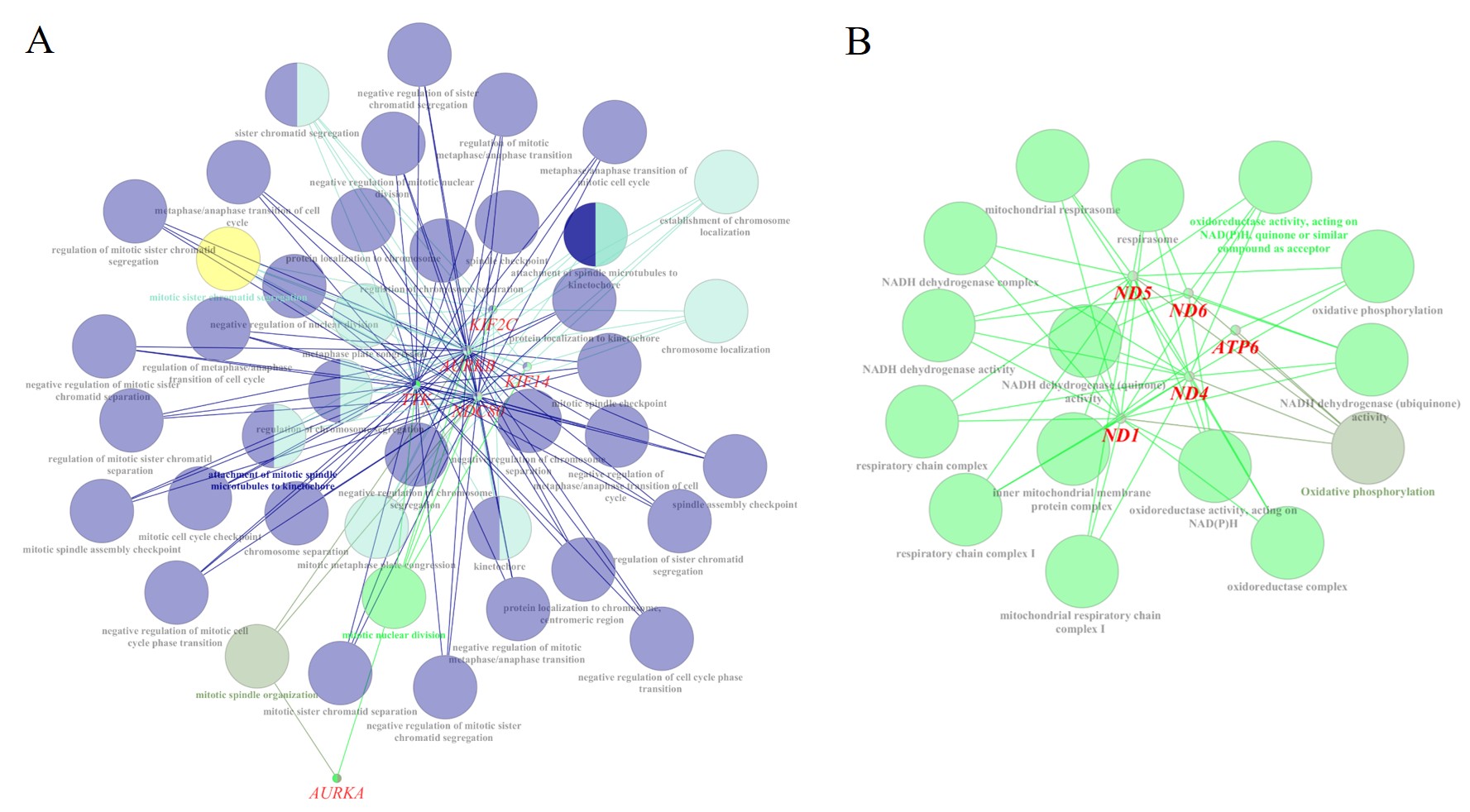


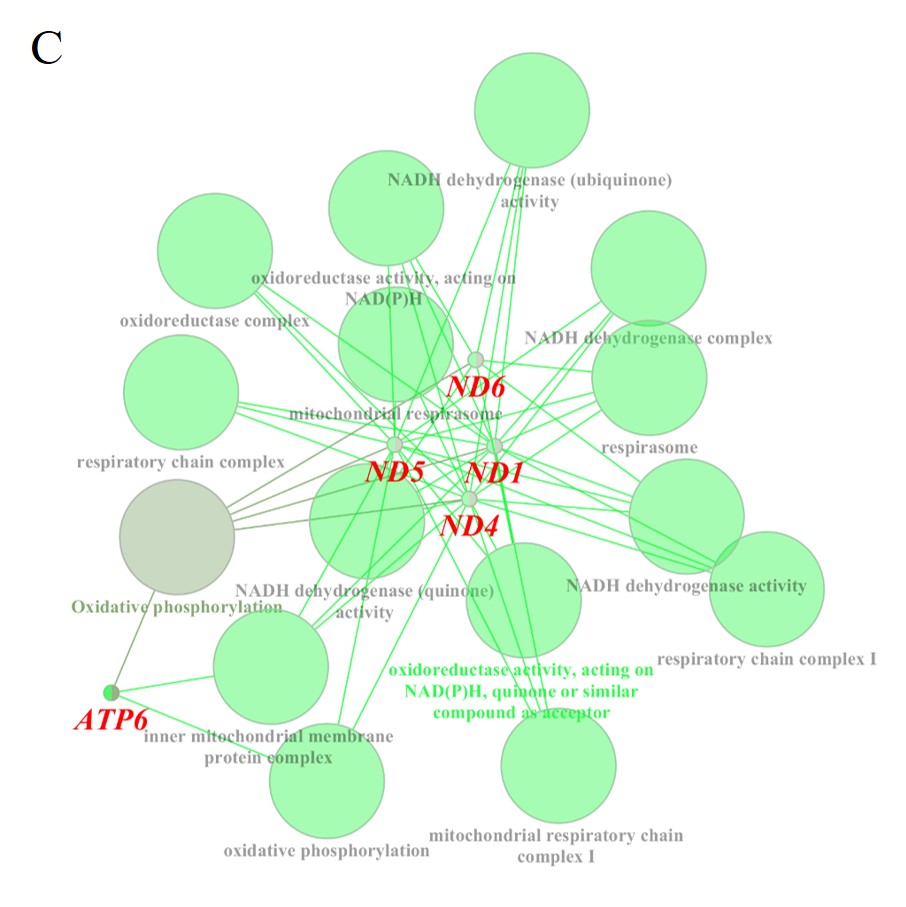


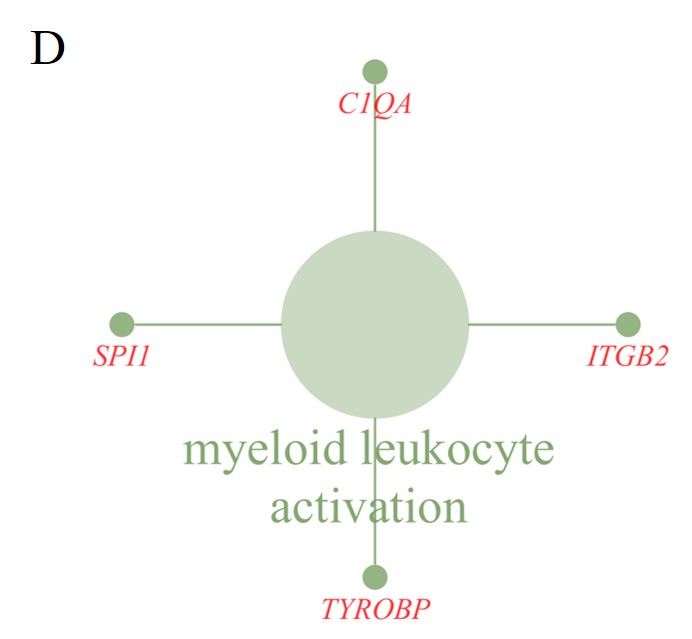


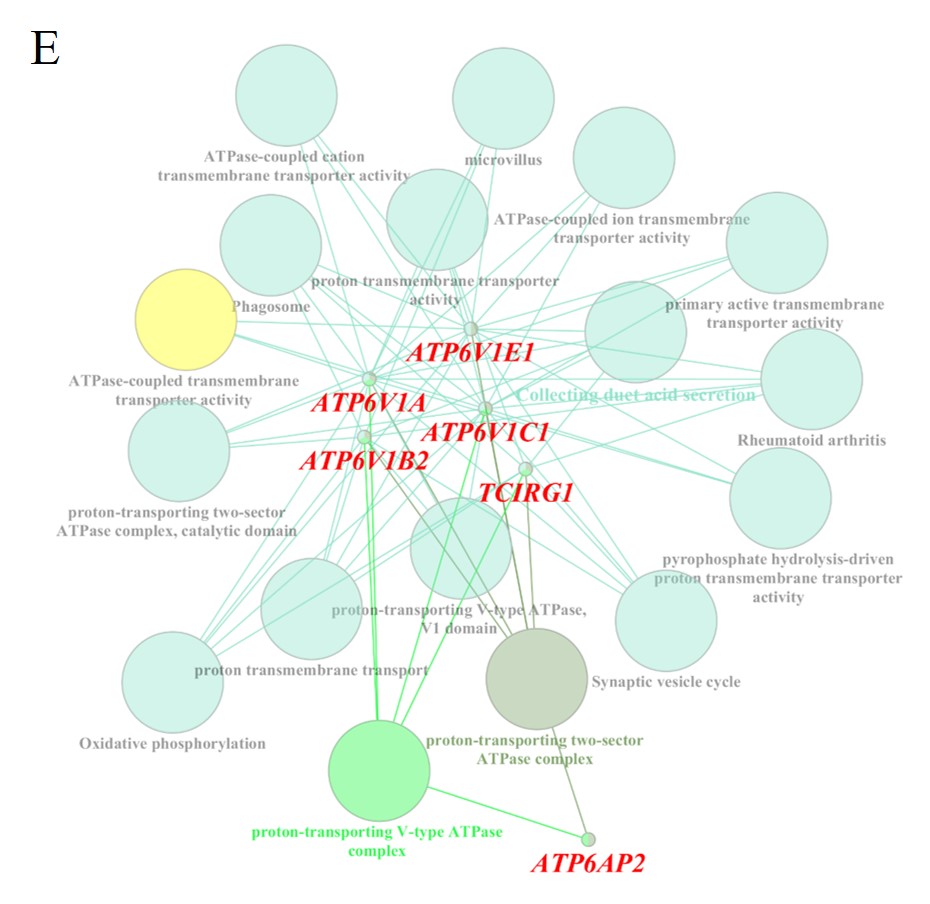


Supplementary Figure 2A-E: Functional enrichment networks. A and B) module 1 and module 2 for LOW *vs* HIGH, C) module 2 for the interaction of prenatal nutrition and sex, D) module 1 for LOW-HCHF and HIGH-HCHF *vs* NORM-CONV and E) module 1 for HCHF *vs* CONV.
